# Supramolecular Polysaccharide Nanotheranostics that Inhibit Cancer Cells Growth and Monitor Targeted Therapy Response

**DOI:** 10.7150/ntno.44703

**Published:** 2020-05-18

**Authors:** Nilesh Deshpande, Anujan Ramesh, Dipika Nandi, Anh Nguyen, Anthony Brouillard, Ashish Kulkarni

**Affiliations:** 1 Department of Chemical Engineering, University of Massachusetts, Amherst, MA, USA; 2 Depatment of Biomedical Engineering, University of Massachusetts, Amherst, MA, USA; 3 Department of Veterinary and Animal Science, University of Massachusetts, Amherst, MA, USA; 4 Center for Bioactive Delivery, Institute for Applied Life Sciences, University of Massachusetts, Amherst, MA, USA

**Keywords:** Polysaccharide, Nanotheranostics, Supramolecular Chemistry, Kinase inhibitor Delivery, Cancer

## Abstract

Targeted anticancer therapies directed against specific molecular drivers of tumors are emerging as effective treatment strategies, however, monitoring their response is still challenging. Current clinical imaging techniques that measure either morphological or metabolic changes in the tumor are not always indicative of clinical outcome due to delayed or variable responses. Here, dual-stage polysaccharide-based supramolecular nanotheranostics (SPN) were designed that enable co-delivery of kinase inhibitor and an activatable imaging probe.

**Methods**: The SPNs were prepared by supramolecular assembly of two components, polysaccharide construct conjugated with kinase inhibitor-function activatable probe and kinase inhibitor- β-cyclodextrin conjugate. Physiochemical characterization of SPNs including size, stability, zeta potential and pH-responsiveness of the assembly was performed. The efficacy of SPNs in inducing cancer cell death by inhibition of kinase signaling and imaging the response was evaluated in murine BRAFV^600E^ melanoma (D4M) and triple-negative breast cancer (4T1) cell lines. Finally, the *in vivo* efficacy was investigated in D4M melanoma tumor model.

**Results**: The polysaccharide-constructs along with kinase inhibitor- β-cyclodextrin conjugates self-assemble to produce SPNs of around 200 nm in diameter and were stable for over a week under physiologically relevant conditions. The SPNs exhibited enhanced cytotoxic effect and significant inhibition of kinase signaling as compared to the free inhibitor. *In vitro* imaging studies confirmed their enzyme-activatable therapy response tracking abilities both in cancer cells and tumor spheroids. Furthermore, SPN treated mice exhibited better tumor growth inhibition as compared to the control groups and therapy response could be imaged at both early (24-48h) and later time points.

**Conclusion**: These findings demonstrate that the supramolecular polysaccharide nanotheranostics can not only inhibit kinase signaling pathway in aggressive tumor, but also monitor targeted therapy response early.

## Introduction

Cancer continues to be one of the major causes of mortality worldwide despite recent advances in cancer therapeutics^1^-^3^. In the past few decades, better understanding of cancer biology has led to the development of molecularly targeted kinase inhibitors that can specifically modulate the functions of one or more proteins which play critical roles in tumor growth^4^-^8^. Cancer progression is mainly driven by the genetic alterations in complex signaling pathways that leads to the activation of specific oncogenes and inactivation of tumor suppressor genes^9^-^12^. Thus, modulating such cellular events offers a way to disrupt tumor growth without significant toxicities. In this context, phosphoinositide 3-kinase (PI3K) and its downstream pathways such as mTOR were found to play a significant role in many physiological processes, such as metabolism, motility, growth and proliferation^13^,^14^. Abnormal activation of this signaling pathway has been discovered in a wide range of human cancers which supports their aggressive growth, making this an attractive target for anticancer therapy^15^-^17^. Remarkable progress has been made in the development of drugs that can modulate these cellular pathways and currently many of these have made their way to clinical trials^18^. However, monotherepeutic agents for inhibition of PI3K pathway including GDC-0941, XL147, PX-866, GDC-0032, IPI-145 and many other have shown limited clinical efficacy^19^-^22^. The diversity among tumors and the intrinsic or acquired resistance possibly limits the clinical efficacies of these small molecule kinase inhibitors 23. In addition, suboptimal tumor accumulation and rapid* in vivo* clearance further contributes towards their lower efficacies 24-26. Therefore, the technology that can not only deliver higher amounts of kinase inhibitor to the tumor but also enable early monitoring of the response to this therapy could help in identifying responsive tumors quickly 27.

Currently nanotechnology-based delivery systems have shown promising results in delivering the therapeutic molecules to the target sites 28. Recently, the combination of therapy and diagnostics termed as “theranostics” has emerged as a smart way of delivering the drugs and imaging agents to target site 29-31. Indeed, nano-assemblies were designed to have imaging markers that enables visualization of tumor accumulation, drug release or its intracellular distribution. However, monitoring drug efficacy in real-time, especially the action of kinase inhibitor in the tumor cells, still remains a challenging task 32-37. The real-time detection of kinase inhibitor efficacy could help in efficient management of cancer where it is crucial to monitor the effect of given treatment on the tumor growth inhibition early 38. Thus, targeting molecular level network such as kinase signaling pathways and monitoring its response at early stages could be a better way of improving therapeutic efficacy through targeted therapies. Unfortunately, most of the kinase inhibitors studied to-date are pharmacologically challenging to administer due to their hydrophobicity 17. Hence, a nanotechnology-based delivery system can offer a better way to administer higher concentration of these inhibitors and protect their pre-mature clearance/degradation 39. In this context, polysaccharide-based biomaterials which are 'designed to degrade after disposal' have been explored as delivery vehicles for a variety of anti-cancer agents including chemotherapy, photodynamic therapy and immunomodulators 40-43. Polysaccharides are naturally occurring polymers with multiple functionalities such as hydroxyl, amine, carboxylic acid, thiols enabling simplicity in chemical modifications 44,45. These intrinsic characteristics and structure of the polysaccharides makes them a suitable material not only to form stimuli-responsive delivery systems but also to carry higher payload of kinase inhibitor and imaging agents together. Integrating polysaccharide-based nanocarriers for molecular level targeting (therapy) with imaging tool that specifically activates upon kinase inhibitor action (diagnostics) would be the ideal way to monitor real-time response of targeted therapy at relatively early stages. However, despite the advancement in the nanotechnology-based kinase inhibitor delivery area, no efforts have been made to design a theranostic delivery vector that can track the efficacy of specific molecular-target therapies.

**Figure 1:**
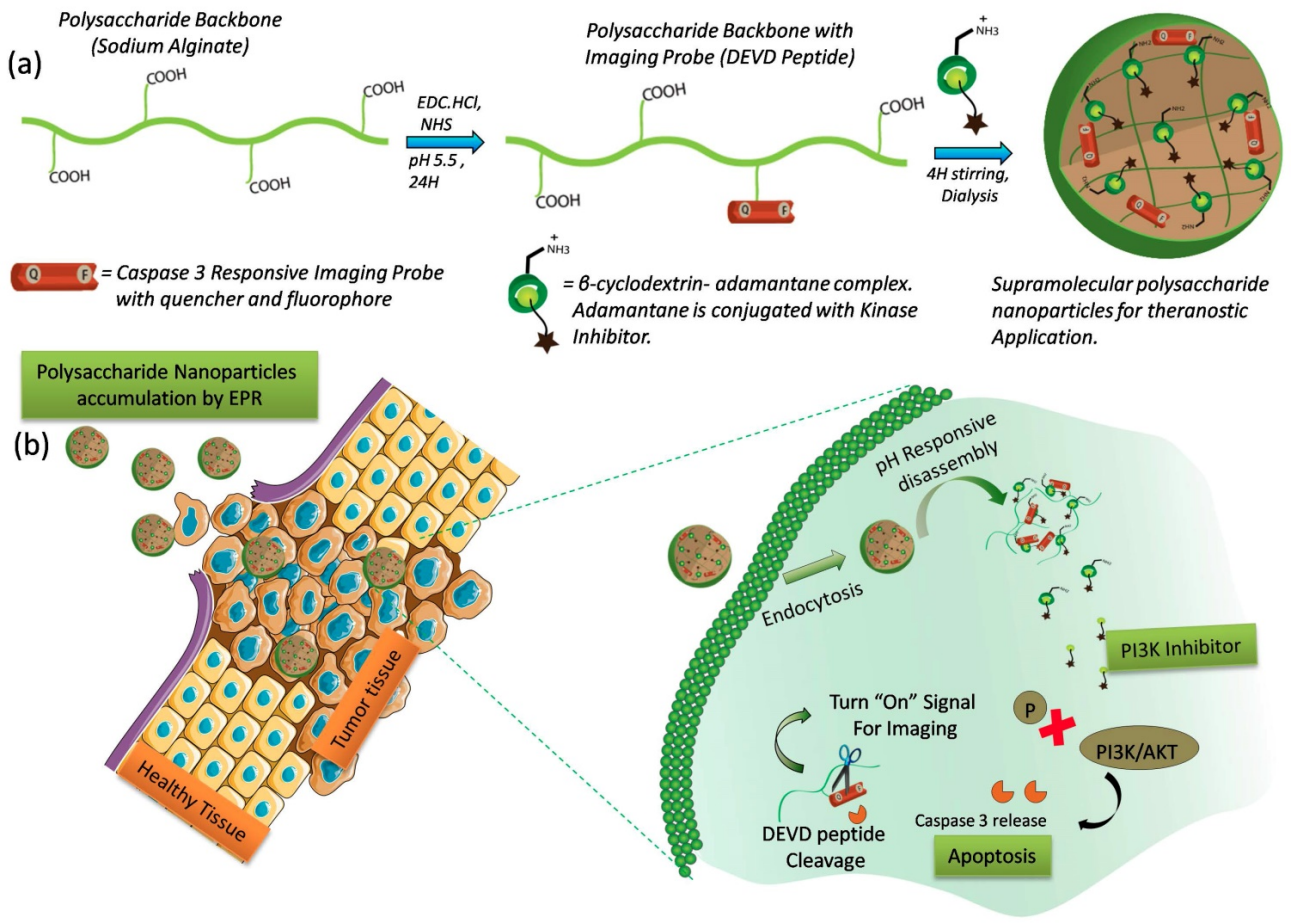
** Design and Mechanism of action of Supramolecular Polysaccharide Nanotheranostics (SPN):** Schematics showing modification of polysaccharide sodium alginate with caspase-3 responsive activatable imaging probe and further encapsulation of β-CD-NH2-kinase inhibitor conjugate using supramolecular approach to form SPN **(a)**, Schematic shows the accumulation of SPNs at the tumor tissue followed by intracellular pH responsive dis-assembly and PI3-kinase inhibitor release **(b)**. The inhibitor induces capsase-3 mediated apoptosis by inhibition of PI3-kinase signaling. The activated enzyme can then cleave the peptide sequence to turn on the imaging signal.

Here, we describe a supramolecular polysaccharide nanotheranostics (SPN) system that enables the co-delivery of PI103, a small molecule PI3K/mTOR inhibitor and a kinase inhibitor-function responsive activatable probe (Figure 1). To ensure prolong circulation time of kinase inhibitor in the bloodstream and to accomplish the target specific release, the SPNs were carefully designed and constructed using two-stage self-assembly approach. In first stage, the activatable probe with peptide sequence (GK-DEVD-APC) and a FRET pair that includes a dye (5FAM) and a quencher (QSY7) on either side of the peptide sequence, was synthesized using a standard solid phase synthesis protocol. This activatable probe was then conjugated to polysaccharide sodium alginate backbone using carbodiimide chemistry at an optimized polymer to peptide ratio to obtain polymer construct. In the second stage, the aqueous solubility of the kinase inhibitor was enhanced by using supramolecular chemistry approach. Briefly, β-cyclodextrin, a water soluble biodegradable host molecule bearing the hydrophobic inner cavity was chosen owing to its ability to form complex with adamantane molecule 46-48. Further, the conjugation of kinase inhibitor (PI103) to adamantane followed by inclusion complexation with β-cyclodextrin, produced a stable cationic water soluble β-cyclodextrin-adamantane-kinase inhibitor complex. Finally, the electrostatic interactions between polymer construct with non-conjugated carboxylic groups and cationic β-cyclodextrin-inhibitor complex facilitated the self-assembly to produce supramolecular polysaccharide nanotheranostics (SPN). The synthesized SPNs were found to be stable for 7 days under the physiologically relevant conditions but were rapidly destabilized at acidic pH as measured by changes in size and zeta potential. The cytotoxic efficacy of the SPNs was demonstrated in resistant melanoma (D4M) and triple negative breast cancer cells (4T1). The SPN's efficacy was also investigated in 4T1 breast cancer 3D spheroids and finally in D4M melanoma tumor mice model. The significant kinase inhibition using SPNs resulted in caspase-mediated apoptosis in cancer cells and activated caspase 3 enzymes then cleaved off the DEVD sequence, releasing the fluorescent dye and activating the fluorescent signal. The activation of the fluorescent dye can be used as a direct way of monitoring the kinase inhibitor activity. SPN's exhibited significant tumor growth inhibition in drug-resistant tumor model as compared to untreated group and demonstrated early kinase inhibitor response monitoring. Thus, the SPN's presents a promising strategy to monitor the efficacy of the targeted therapies at early stages.

## Experimental Section

**Materials and Methods:** Sodium alginate, 4-dimethylaminopyridine (DMAP), 4-toulenesulfonylchloride, ethylenediamine, calcium chloride, sodium carbonate, Tris buffer, (3-(4,5-dimethylthiazol-2-yl)-2,5-diphenyltetrazolium bromide) (MTT), triethylamine (TEA), and precoated silica gel aluminium sheets 60 F254 were purchased from Sigma-Aldrich and used as it is without further purification. N-hydroxy succinamide (NHS), succinic anhydride, phenolphthalein, sodium chloride was purchased from fisher scientific. Adamantane methanol, β-cyclodextrin, and 1-ethyl-3-(3-dimethylaminopropyl) carbodiimide hydrochloride (EDC.HCl), DMSO-d6 and CDCl_3_ were purchased from Acros Chemicals. Dichloromethane (DCM), acetone, N, N dimethylformamide (DMF), dimethyl sulfoxide (DMSO), absolute ethanol, ethyl acetate, pet ether and methanol were brought from Fisher Scientific. NHS-fluorescein and PI-103 were purchased from thermo scientific and Selleck chemicals respectively. All the other chemicals and solvents used were purchased from fisher scientific. Dulbecco's modified eagle medium (DMEM), fetal bovine serum (FBS) and antibiotics were purchased from Gibco, life technologies. Primary rabbit anti mouse cleaved caspase 3 antibody and Alexa Fluor 594 goat anti rabbit IgG secondary antibody were purchased from cell signaling technologies. Recombinant caspase-3 were purchased from Abcam and used as per the manufacturer's protocol. Nanoparticles size and zeta potential were recorded on Malvern Zetasizer ZSP equipped with 630nm red laser. UV-Vis absorbtion spectra were recorded on Shimazdu UV3600 spectrophotometer. Transmission electron microscopy (TEM) was done using FEI Technai Cryo-Bio 200kV FEG TEM instrument. The samples were drop casted on copper grid at concentration of 0.05mg/mL and dried overnight under vacuum. The fluorescence measurements were carried out on Biotek plate reader. For MTT assay the absorbance measurements were carried out on Biotek plate reader and data is plotted using GraphPad Prism. The confocal microscopy images were captured on Nikon A1SP. NMR measurements were carried out on 400 MHz Bruker instrument using tetramethyl silane as internal standard. The data was analyzed using ACD NMR academia edition software.

**Synthesis of 4-(((3r,5r,7r)-adamantan-1-yl) methoxy)-4-oxobutanoic acid (a):** Adamantane methanol (1.0 g, 6.02 mmol) and DMAP (0.73 g, 6.02 mmol) were dissolved in 10.0 mL of dichloromethane and stirred for 15 minutes at 25^0^C. To above reaction mixture, succinic anhydride (0.72 g, 7.22 mmol) was added and resulting mixture was stirred at 25^0^C for 12 hours. DCM in the reaction mixture was removed using vacuo evaporation, obtained solid was dissolved in acidic water (pH 5.0) and the product was extracted using DCM. The organic layer was dried over anhydrous magnesium sulfate, evaporated to obtain white solid as a pure product. Yield = 1.3 g (81%): ^1^H NMR (500 MHz, CHLOROFORM-*d*) δ ppm : 1.48 - 1.57 (m, 6 H) 1.65 (d, *J*=11.90 Hz, 3 H) 1.74 (d, *J*=12.21 Hz, 3 H) 1.99 (br. s., 3 H) 2.64 - 2.74 (m, 4 H) 3.02 (s, 1 H) 3.71 (s, 2 H) (See Supplementary Figure 4).

Synthesis of ((3r,5r,7r)-adamantan-1-yl)methyl (3-(4-morpholinopyrido[3',2':4,5]furo[3,2-d]pyrimidin-2-yl)phenyl) succinate (AD-SA-PI103) (b): PI-103 (10 mg, 0.028 mmol) and Compound (a) (7.64 mg, 0.028 mmol) were dissolved in 1.0 mL of N,N dimethylformamide. To the above mixture, EDC.HCl (8.25 mg, 0.043 mmol) and DMAP (1.05 mg, 0.008 mmol) were added and the resulting mixture was stirred at 25^0^C for 24 hours. The DMF in the reaction mixture was removed by lyophilization, obtained solid contents were dissolved in water and the product extracted using DCM. The obtained organic layer was dried over anhydrous magnesium sulfate, evaporated to yield white solid as a crude product. The pure product is obtained by passing over silica (100-200 mesh) using 2% methanol: dichloromethane solvent system. Yield = 13 mg (76%).^1^H NMR (400 MHz, CHLOROFORM-*d*) δ ppm 1.49 - 1.61 (m, 6 H) 1.66 (br. s., 3 H) 1.71 (br. s., 3 H) 1.99 (br. s., 3 H) 2.77 - 2.86 (m, 2 H) 2.93 - 3.00 (m, 2 H) 3.75 (s, 2 H) 3.88 - 3.96 (m, 4 H) 4.20 - 4.28 (m, 4 H) 7.18 - 7.26 (m, 1 H) 7.44 - 7.56 (m, 2 H) 8.17 - 8.25 (m, 1 H) 8.31 - 8.46 (m, 1 H) 8.51 - 8.63 (m, 2 H) (See Supplementary Figure 9).

**Synthesis of 4-((((3r,5r,7r)-adamantan-1-yl)methoxy)carbonyl)-2-(6-hydroxy-3-oxo-3H-xanthen-9-yl)benzoic acid (AD-FITC) (c):** Adamantane methanol (10 mg, 0.06 mmol) and DMAP (7.35 mg, 0.06 mmol) were dissolved in 2.0 mL of DMF and stirred for 30 minutes at 25^0^C. To above mixture, fluorescein-NHS (28 mg, 0.06 mmol) was added and the resulting solution is stirred at 25^0^C for 24 hours. DMF from the reaction mixture is removed by lyophilization, obtained solid content is dissolved in water and the product is extracted in ethyl acetate. The obtained organic layer is dried over anhydrous magnesium sulphate to yield a yellowish green solid as a crude product. The crude product is purified by passing over silica (100-200 mesh) using 5% methanol: DCM as an eluent. Yield = 20 mg (64%).^1^H NMR (400 MHz, CHLOROFORM-*d*) δ ppm 0.99 - 1.19 (m, 3 H) 1.19 - 1.36 (m, 3 H) 1.37 - 1.56 (m, 15 H) 1.60 (br. s., 5 H) 1.67 (br. s., 11 H) 1.70 - 1.80 (m, 12 H) 1.99 (br. s., 11 H) 2.46 - 2.74 (m, 4 H) 3.21 (s, 1 H) 3.47 (d, *J*=5.38 Hz, 2 H) 3.51 - 3.81 (m, 6 H) 3.84 - 4.12 (m, 2 H) 6.60 (br. s., 1 H) 6.67 - 6.79 (m, 2 H) 6.82 (br. s., 1 H) 6.87 - 7.07 (m, 1 H) 8.15 - 8.43 (m, 1 H) (See Supplementary Figure 10).

**Synthesis of β-CD-OTS (d):** β- cyclodextrin (1 g, 0.88 mmol) and 4-toulene sulfonyl chloride (0.4 g, 2.10 mmol) were dissolved in 50 mL of water and stirred for 2 hours at 25^0^C. To the above solution, 1 g of sodium hydroxide was added and stirred for another 10 minutes. The formed turbid reaction mixture was vacuum filtered, and filtrate was made acidic (pH= 6.5) using ammonium chloride. The obtain white turbid solution was kept at 4^0^C for 12 hours and the resultant white precipitate was separated by filtration, washed several times with acetone, dried and stored at 4^0^C. Yield = 0.7 g (62 %)^1^H NMR (500 MHz, DMSO-*d*_6_) δ ppm 2.09 (s, 2 H) 2.43 (s, 3 H) 2.51 (d, *J*=1.83 Hz, 15 H) 3.22 - 3.35 (m, 21 H) 3.38 (br. s., 6 H) 3.48 - 3.70 (m, 22 H) 4.06 - 4.61 (m, 7 H) 4.76 - 4.86 (m, 6 H) 5.63 - 5.84 (m, 12 H) 7.35 - 7.50 (m, 2 H) 7.76 (d, *J*=8.24 Hz, 2 H) (See Supplementary Figure 5).

**Synthesis of β-CD-NH_2_ (e):** Compound (d) (0.5 g, 0.38 mmol) was dissolved in 2.0 mL of ethylene diamine and stirred at 80^0^C for 48 hours. The reaction mixture was cooled to 25^0^C and poured in excess volume of acetone to obtain a white precipitate which was separated by filtration. The obtained white precipitate was washed several times with acetone, dried and stored at 4^0^C. Yield = 0.35 g (77%) ^1^H NMR (500 MHz, DMSO-*d*_6_) δ ppm 2.55 - 2.89 (m, 4 H) 3.05 - 3.43 (m, 26 H) 3.48 - 3.80 (m, 29 H) 4.82 (br. s., 8 H) 4.69 - 4.92 (m, 8 H) (See Supplementary Figure 5).

**Synthesis of caspase 3 enzyme activatable peptide (GK-DEVD-APC) activatable probe (f):** The synthesis of activatable probe was performed as previously described^49^. Briefly, the peptide sequence was synthesized using standard Fmoc solid phase synthesis protocol and the formed peptide was charecterised using MALDI-TOF. The amine functionality of the lysine amino acid was functionalized with FITC-NHS (fluorophore) using carbodiimide chemistry and cysteine residue was functionalized with a quencher molecule (QSY7). The formed peptide was purified using HPLC and charecterised using MALDI-TOF technique.

**Synthesis of activatable probe modified sodium alginate derivative (Polymer construct) (g):** sodium alginate (5 mg, 0.028 mmol) was dissolved in 2.0 mL of water by stirring it for 15 minutes. Then to above solution, NHS (4.90 mg, 0.042 mmol) and EDC were added, and pH of the resulting solution is maintained as 5.0 using tris buffer. Meanwhile GK-DEVD-APC peptide (1.5 mg, 0.00068 mmol) having fluorophore and quencher were dissolved in 500μL of DMSO and added to sodium alginate solution and reaction was continued for 24 hours at 25^0^C. The product is extracted by using precipitation technique, wherein the reaction mixture was dropped slowly in excess amount of ethanol and the obtained precipitate was separated by filtration. This product is purified by reprecipitation technique, finally dissolved in water and lyophilized to yield dark violet color solid compound. Yield = 3.7 mg. The substitution of activatable probe on polysaccharide backbone was confirmed by ^1^H NMR and the amount of probe present on the polysaccharide backbone was calculated by UV-VIS absorbtion spectroscopy using Lambert-Beers law. ^1^H NMR (400 MHz, DEUTERIUM OXIDE) δ ppm 0.74 - 1.32 (m, 2 H) 1.67 - 1.93 (m, 1 H) 2.69 (s, 8 H) 2.77 - 2.85 (m, 1 H) 2.93 - 3.23 (m, 2 H) 3.61 - 3.75 (m, 2 H) 3.80 - 4.11 (m, 3 H) 6.76 - 6.90 (m, 1 H) 7.83 - 8.26 (m, 1 H) 8.31 - 8.49 (m, 1 H).

Following the above procedure, sodium alginate derivative without a quencher molecule was synthesized and used as a fluorescent control for cellular uptake experiments.

**Synthesis of β-cyclodextrin-adamantane-kinase inhibitor/dye Inclusion Complexes:** β-cyclodextrin-NH_2_ (1.0 mg, 0.0008 mmol) was dissolved in 1.0 mL of milli-q water and stirred for 10 minutes. Adamantane-PI103 (0.50 mg, 0.0008 mmol) conjugate was dissolved in 200 μL of DMSO and mixed with β-CD-NH_2_ solution. The resulting solution was sonicated for 5 minutes and lyophilized to obtain the inclusion complex. The inclusion complex formation was confirmed by selective 1D ROESY NMR experiment and competitive phenolphthalein binding assay experiment as described in the upcoming section. This complex was stored at 4^0^C. Using similar protocol, β-CD-NH_2-_ adamantane-FITC complex was made and stored at 4^0^C.

**Synthesis of supramolecular polysaccharide nanotheranostics (SPN):** Polysaccharide nanotheranostics were made by dissolving 1:1 weight ratio of modified sodium alginate (g) and β-CD-NH_2_- inclusion complex (kinase inhibitor/dye) in milli-q water followed by 4 hours of stirring at 25^0^C. The resulting solution was dialyzed against milli-q water for 24 hours, and then lyophilized. The obtained solid powder was stored at 4^0^C and used for further studies. To make control polysaccharide nanotheranostics (without the kinase inhibitor molecule, PI103), only β-CD-NH_2_ was used instead of kinase inhibitor/dye inclusion complex. To study intracellular biodistribution, nanoparticles were made using sodium alginate derivative which do not have quencher molecule. The sizes and zeta potential of the SPN were monitored by dynamic light scattering technique.

**Determination of kinase inhibitor/dye concentration in SPN:** UV-Vis absorbtion spectroscopy was employed for determination of kinase inhibitor/dye concentration. 0.5 mg of SPN was dissolved in 500 ul of milli-q water and the absorbance measurement was done at 500 nm using Shimazdu UV3600 instrument. A standard curve was generated using different concentrations of free kinase inhibitor/dye/peptide and equation obtained was used to determine the concentration of kinase inhibitor/dye molecules present in the SPN.

**Stability studies of SPN:** The stability of SPN was monitored by measuring the change in the hydrodynamic radius and zeta potential of SPN by using dynamic light scattering technique with Malvern Zetasizer ZSP instrument equipped with 630nm red laser. SPNs were incubated with human serum, water and phosphate buffer saline at 4^0^C at concentration of 0.05 mg/mL and average hydrodynamic diameter and zeta potential was measured over seven days.

**Phenolphthalein-β-CD-NH_2_-adamantane competitive binding assay:** UV-Vis absorbtion spectroscopy was employed to study this competitive binding assay experiment. Phenolphthalein stock solution was made in water and pH= 10.5 was adjusted using 0.1M sodium hydroxide solution. An equal volume of phenolphthalein solution (0.42 mM) were transferred to different vials and titrated against various concentrations (0.21 mM to 2.12 mM) of β-CD-NH_2_ solutions in water until end point appears from pink to colorless. To study the competitive binding effect of adamantane on the phenolphthalein-β-CD-NH_2_ complex, 0.42 mM of adamantane-succinic acid was added to the complex solution in last vial and color change was monitored. To record the UV-Vis spectrum of these solutions, 20 ul solutions was further diluted to 200 ul with basic water and measurements were done. A plot of absorbance change at 554 nm against the concentration of β-CD-NH_2_ was generated and the competitive binding was studied. All the solution preparation and measurements were carried out on the same to avoid any error in the experiment.

***In vitro* caspase 3 susceptibility of activatable imaging probe:** Fluorescence spectroscopy was employed to study enzyme susceptibility towards the activatable probe activation. 5μM of GK-DEVD-APC activatable probe conjugated with fluorophore (5FAM) and quencher (QSY7) was incubated with 5U of recombinant caspase 3 enzyme in 200μL of caspase assay buffer at 37^0^C and change fluorescence intensity at 525nm was monitored using Biotek microplate reader at different time intervals ranging from 0-6 hours. A control was also included where the activatable probe was incubated in caspase assay buffer without caspase 3 enzyme. A plot of fluorescence intensity versus time was generated using origin software to analyze the vulnerability of peptide cleavage. A similar experiment was performed with SPN to study the activity of caspase 3 enzyme in cleaving the peptide sequence when present in polysaccharide nanoparticles form.

**Cytotoxic efficacy of SPN's using MTT assay:** To study the cytotoxic effects of the SPN's and their kinase inhibitor loaded counterparts, 5 × 10^3^ cells (D4M/ 4T1) per well were seeded in 96-well plate and allowed to adhere for 16-18 hours. The complete DMEM media from the wells were aspirated and cells were treated with new media having different concentration of SPN, kinase inhibitor loaded SPN's and free kinase inhibitor for 48 hours. After 48 hours, the media containing kinase inhibitors were aspirated and cells were treated with fresh media having MTT reagent (0.5mg/mL) and incubated for another 4hrs. The formed formazan crystals were dissolved in 100μL of DMSO and the absorbance at 570 nm was monitored using Biotek 96 well plate reader. The data represents the values from at least three independent measurements. The relative percentage values with respect to control were calculated and plotted against the concentration to assess the cell viability. The doubling times for D4M and 4T1 cells were 16 and 22 hours respectively. The 4T1 cells were obtained from ATCC with the original strain BALB/cfC3H. The D4M cells were a gift from D. Fisher, Massachusetts General Hospital, Boston, MA.

**Intracellular biodistribution of the SPN's in cancer cells:** To study the cellular internalisation of the SPN's, 1 × 10^5^ cells were seeded on a cover slip placed in 6 well plate in complete DMEM an allowed to adhere for 16-18 hours. After 18 hours, the cells were treated with fresh media having the required kinase inhibitor concentration (5μM) and incubated for different time points as needed. Two controls were also included where the cells were treated with SPN's without kinase inhibitor and SPN's without quencher molecule to study the relative cellular internalisation and effect of the kinase inhibitor on activation of the fluorescence signal of the SPN's in intracellular environment. After required time of incubation, the cells were washed thoroughly with 1× PBS to remove unwanted impurities. Cells were then fixed with 4% paraformaldehyde, stained with DAPI and mounted on a glass slide using invitrogen antifade reagent. The slides were dried and imaged using confocal microscope.

**Western blot analysis to study PI3K inhibition using SPN:** To study the PI3K inhibition using western blot, 2 × 10^5^ cells were seeded in each well of 6 well plate and allowed to adhere for 16-18 hours. After 18 hours, the cells were treated with required concentration of free kinase inhibitor and kinase inhibitor loaded SPNs and incubated for desired time points. After incubation for optimum time point, cells were lysed using RIPA lysis buffer with protease and phosphatase inhibitor. The amount of protein was calculated by standard BCA assay protocol, and equal amount of protein lysates were electrophoresed on a 10% polyacrylamide gel and transferred to PVDF membrane followed by incubation with phospho-Akt (1:1000 dilution), total-Akt (1:1000 dilution) and β-actin (1:2000 dilution) antibodies for overnight at 4^0^C. After washing with TBST, membranes were incubated with horseradish peroxidase conjugate secondary antibody (1:2000) for 1 hour. The detection was carried out using Biorad's clarity ECL, substrate images were taken using Biorad's Chemidoc and processing was done by image J software.

***In vivo SPN efficacy in D_4_M mouse model:***To study the *in vivo* efficacy of SPN, D4M cells (1× 10^6^) were injected subcutaneously into the right flank of the C57B/L6 mice and tumors were allowed to grow. After 10 days of tumor inoculation (tumor size of ≈ 50-75 mm^3^), mice were randomly divided into three groups (n =3) and the start of the treatment is referred as day 0. The treatments of (I) vehicle, (II) free kinase inhibitor + SPN without kinase inhibitor and (III) SPN with kinase inhibitor (PI103) were given intravenously at concentration of 3 mg/kg of kinase inhibitor and 1 mg/kg of the imaging probe. The treatments groups were injected to the mice intravenously in the tail vein with the volume of each injection being around 200 μL. The volume of the injections was used in order to maintain the kinase inhibitor and imaging probe concentration as 64 and 21 μg per injection respectively. The mice were restrained using mouse restrainer during the treatment. These injections were administered on every day from 0-5 days and the tumor volume was monitored on every alternate day until day 10. Tumor dimensions were measured using Vernier caliper and the tumor volumes were calculated using the formula ((L×B^2^)/2), where L is considered as the longest diameter and s is considered to be the shortest diameter. Mice were sacrificed when tumors either became necrotic or reached the size of around ~2000 mm^3^. After sacrificing mice, the excised tumor tissues were used to study the kinase inhibitor efficacy after ten days of drug administration. The fixing of the tumor sections is discussed in the *ex vivo* imaging section. All the protocols followed during the studies were approved and in accordance with guidelines provided by the institutional animal care and use committee (IACUC) of the University of Massachusetts Amherst.

***Ex vivo efficacy of SPN in D_4_M tumor model:***To capture the *ex vivo* efficacy of the SPN using the imaging probe, D4M cells (1× 10^6^) were injected subcutaneously into the right flank of the C57B/L6 mice and tumor were allowed to grow. After 10 days of tumor inoculation (tumor size of ≈ 75mm^3^), mice were divided into three groups (n=2) and treatment was started and referred as day 0. The treatment of (I) vehicle, (II) free kinase inhibitor + blank SPN and (III) SPN with kinase inhibitor (PI103) were given intravenously at concentration of 3 mg/kg of kinase inhibitor and 1 mg/kg of the activatable probe to each group. After 24 and 48 hours of treatment, the mice were sacrificed, and the tumors were isolated. To image the kinase inhibitor sensitivity, tumor sections of 5μm were cut using cryostat and then fixed using 4% paraformaldehyde. To image the cleaved caspase 3, the tumor sections were stained with primary rabbit anti mouse cleaved caspase 3 antibody followed by Alexa flour 594 goat anti rabbit IgG secondary antibody. The nuclei in the tumor tissues were stained with DAPI and the imaging was performed with Nikon A1R-SIMe confocal microscope at ×20. The obtained data is processed using NIS element software.

**Determination of PI103 inhibitor concentration in cells treated with SPN:** In typical experiment, the D4M cells were seeded in six well plates at density of 10^5^ cells per well and allowed to adhere for 18 hours. Cells were then treated with 10μM concentration of SPN for different time points such as 18, 12 and 6 hours. After the successive treatment, the cells were washed thrice with 1X PBS and then treated with 1.0 mL of dimethylsulfoxide for 2 hours to facilitate the lysis of the cells. After two hours, the cells were removed using scrapper and then centrifuged to remove the cell pellet. The supernatant DMSO was collected in separate vial and used for absorbance measurement. The concentration of the PI103 inhibitor in thee lysate was calculated using the standard equation given in supplementary figure. Cells without SPN treatment were used as control in this experiment.

**TUNEL Assay:** The stored tissues from different treatment groups were frozen in OCT (Tissue Tek) and sectioned into 5 uM thin slices and mounted over the glass slides. Tissue sections were stained with Alexa Fluor 588 Click -iTTM Plus TUNEL Assay Kit (Thermo Fisher) as per the manufacturer's protocol. Nuclei were stained with DAPI (Blue). Imaging was performed with Nikon A1R-SIMe confocal microscope at 20x and analyzed with NIS Elements 4.6.

## Result and Discussion

***Synthesis of polymer construct bearing activatable imaging probe:*** A supramolecular interactions assisted two-stage nanocarriers platform was designed and synthesized using a natural resource-based polysaccharide sodium alginate. This platform delivers otherwise water insoluble targeted kinase inhibitor to the intracellular milieu to achieve the desired kinase inhibition effects and also monitors the intracellular kinase inhibitor response using fluorescent tracking probe. The construction of the nano-assembly includes a two-step self-assembly process. First step involves the synthesis of polymer construct bearing activatable probe that monitors the kinase inhibitor response. The polymer construct employed in the present system facilitates the stimuli responsive release of the contents at the target site. In second step, bioavailability of the kinase inhibitor was enhanced by using β-cyclodextrin-adamantane supramolecular chemistry approach. Pre-conjugation of kinase inhibitor (PI103) with adamantane followed by inclusion complexation with β-cyclodextrin, produced a stable cationic water soluble β-cyclodextrin-adamantane-kinase inhibitor complex. Finally, the electrostatic interactions between polymer construct having free carboxylic groups and cationic β-cyclodextrin-inhibitor complex facilitated the self-assembly to produce supramolecular polysaccharide nanotheranostics (SPN). To construct such a design, a biocompatible and biodegradable sodium alginate backbone was chosen owing to its potential clinical utility and ease of functionalization. The presence of free carboxyl functionalities on polysaccharide backbone provides an opportunity to synthetically customize sodium alginate chain which can direct the aqueous self-assembly of the newly synthesized polysaccharide derivatives. Current approach employs free carboxyl groups on sodium alginate to chemically conjugate the activatable probe that enables the fluorescence-based tracking of the kinase inhibitor response. The peptide sequence (GK-DEVD-APC) was designed to exclusively cleave upon the kinase inhibitor action with release of activated caspase 3 enzyme which will specifically degrade the DEVD part of the peptide. A fluorophore (5FAM) and quencher (QSY7) molecules were conjugated on either side of DEVD peptide sequence to induce emission turn-off signal based upon fluorescence resonance energy transfer phenomenon 49. Fluorescence activation upon the peptide cleavage allows the real time monitoring of the kinase inhibitor action in the intracellular environment. The amine group on the one end of peptide was coupled on carboxyl group of the polysaccharide backbone using carbodiimide chemistry as shown in the synthetic scheme in **Supplementary Figure 1 and 2**. The substitution of activatable imaging probe on polysaccharide backbone was optimized using different polymer to activatable probe ratios and finally the ratio of sodium alginate to activatable probe was kept constant as 1:0.03. With this ratio, the formed sodium alginate derivatives were found to be dispersible in water to drive the self assembly to form supramolecular complexes. The amount of peptide or dye molecule conjugated to the polysaccharide backbone was calculated by measuring the absorbance of the peptide or dye when conjugated to polymer and compared with the standard curve (**Supplementary Figure 3**). The amount of peptide present on polymer backbone was calculated to be ~30μg of peptide per mg of polymer.

**Figure 2:**
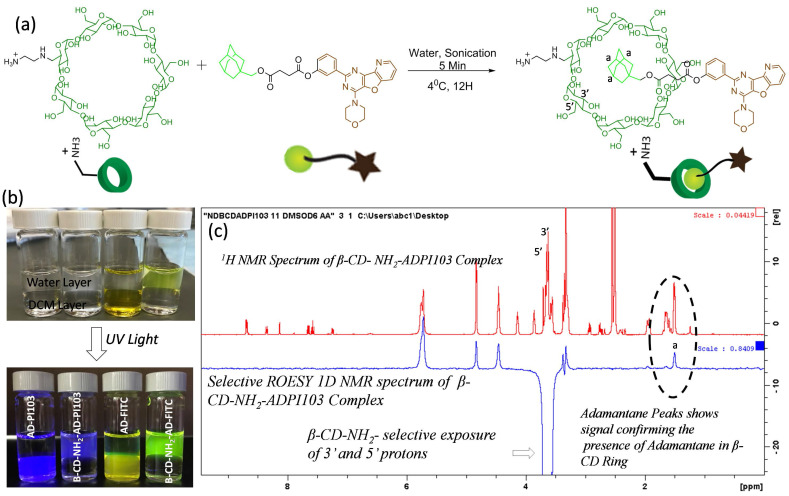
** Synthesis and characterization of PI103- β-cyclodextrin** (β-CD)** inclusion complex:** Synthetic scheme showing inclusion complexation between β-CD-NH_2_ and adamantane-PI103 conjugate (a), Sample vials showing the stabilization of adamantane-kinase inhibitor/dye conjugates inclusion complexes with β-CD in aqueous layer (b), and selective 1D ROESY NMR spectrum of β-CD-NH_2_- and ADPI103 complex confirming the inclusion complex formation (c). The photographs of the vials are taken in daylight as well as in UV light where the bottom layer is of dichloromethane and top layer is water. The inclusion complexation of β-CD-NH_2_- ADPI103 (vial 2) and β-CD-NH_2_- AD-FITC (vial 4) confirms the stabilization of adamantane kinase inhibitor/dye complexes in water. The selective 1D ROESY spectrum is recorded using d6-DMSO as a solvent. The selective exposure of 3' and 5' protons of β-CD-NH_2_ and corresponding appearance of adamantane proton (a) confirms the inclusion complex formation.

***Formation of host-guest inclusion complexes with β-CD-NH_2_ and adamantane- kinase inhibitor conjugate:***To increase the bioavailability of the water insoluble small molecule inhibitor PI103 and to ensure the sustained release of PI103 from the SPNs, a β-cyclodextrin-adamantane inclusion complexation approach was employed. Adamantane with an acid functional group and β-cyclodextrin with a single amine group were synthesized and characterized using ^1^H NMR (**Supplementary Figure 4 and 5**). The amine functional group at 6'-position of β-cyclodextrin can have electrostatic interactions with acid groups on the modified polymeric sodium alginate backbone. These interactions drive the self-assembly of the sodium alginate derivatives to form supramolecular polysaccharide nanotheranostics. Adamantane acid derivative was further modified with kinase inhibitor or dye molecules using robust carbodiimide coupling chemistry (**Supplementary Fig. 9 and 10**). The inclusion complexation of β-cyclodextrin-NH_2_ and adamantane-PI103 was carried out by ultrasonication as shown in the **Figure 2a** and the formed complexes were immediately freeze dried. Using similar protocol, a β-cyclodextrin-NH_2_ and adamantane FITC complexes were also synthesized. (**Supplementary Figure 6**). To confirm the complex formation, free adamantane-PI103 (AD-PI103) and adamantane-FITC (AD-FITC) derivatives were partitioned between dichloromethane and water layer, and as expected both AD-PI103 and AD-FITC retains in the organic layer (**Figure 2b**) owing to their aqueous insolubility. Upon the addition of water soluble β-cyclodextrin-NH_2_ to the same solution and gentle shaking_,_ the AD-PI103 and AD-FITC immediately partitioned in the aqueous layer. This confirms the formation of β-cyclodextrin-NH_2-_adamantane inclusion complexes (**Figure 2b**). These complexes can stabilize water insoluble compounds in the aqueous microenvironment and could be the best vectors to transport kinase inhibitors across the cell membrane. To further confirm the formation of inclusion complexes, A ^1^H NMR experiment of AD-PI103 and β-cyclodextrin-NH_2-_adamantane-PI103 was performed (**Supplementary Figure 7**) where the proton “a” of the adamantane group showed a little downfield shift and a change in the splitting pattern which confirms the complex formation. The obtained results are in accordance with literature reports where similar observations have been made^46^-^48^ To further validate the inclusion complex formation, a selective 1D ^1^H-^1^H rotating frame overhouse effect spectroscopy (ROESY) NMR experiment was performed for β-cyclodextrin-NH_2-_adamantane-PI103 complex (**Figure 2c**). To study the interactions of 3' and 5' protons of β-cyclodextrin-NH_2_ with protons 'a' of the adamantane, a selective exposure of 3' and 5' protons were done using ROESY experiment and the appearance of corresponding adamantane protons 'a' validated the presence of adamantane moiety in the core of β-cyclodextrin-NH_2._ The presence of 3' and 5' protons towards the more hydrophobic side of the β-cyclodextrin-NH_2_ core makes these interactions feasible. The appearance of peaks at 4.2-5.9 ppm in selective ROESY spectrum corresponds to the β-cyclodextrin-NH_2_ protons which are present in the proximity of 3' and 5' protons and hence showed a positive signal. Overall this selective 1D NMR experiment enlightens the interactions between different protons when in close proximity, validating the complexation phenomenon. These experiments provided both visual (**Figure 2b**) and spectroscopic (**Figure 2c**) validation of host-guest complex formation.

***Competitive binding assay for quantitative assessment of host-guest interaction:***The quantitative aspect of β-cyclodextrin-NH_2_-adamantane complexation was studied using phenolphthalein binding assay. The assay utilizes the sensitivity of phenolphthalein towards the hydrophobic environment and affinity of adamantane towards complexation^50^. Briefly, phenolphthalein is a dye molecule and shows a pink color in the basic pH (10.5) due to the delocalization of the electrons in the chemical structure. When occupied in hydrophobic pocket of the β-cyclodextrin-NH_2,_ delocalization of the electrons is arrested due to the lactonization of the ring and thus, the absorbance decreases and resulting in reduction in the pink color. The schematics of the experiments are showed in **Supplementary Figure 8** that explains the sequence of steps performed during this experiment. A fixed concentration of phenolphthalein (0.22 mM) was used and the absorbance was measured after titration against different concentrations of β-cyclodextrin-NH_2_ **(Supplementary Figure 8b)**. The plot clearly shows that with increasing concentration of β-cyclodextrin-NH_2,_ the absorbance of phenolphthalein decreased and completely disappeared at a concentration of 2.12 mM. The addition of adamantane-succinic acid at this point, replaces the phenolphthalein in the β-cyclodextrin-NH_2_ core and thus, phenolphthalein regains its absorbance due to delocalization of electrons **(Supplementary Figure 8c)**. A bar plot showing the decrease in absorbance intensity against β-cyclodextrin-NH_2_ concentration was generated which dictates the quantitative information about β-cyclodextrin-NH_2_ concentration being utilized and the adamantane concentration needed for replacement of phenolphthalein from the core. The replacement of phenolphthalein from the β-cyclodextrin-NH_2_ core was attributed to strong association constant (Ka) of β-cyclodextrin- adamantane complexes as well explored in the literature 50. Thus, from the above experiments and the results obtained from NMR experiments strongly supports the formation of the inclusion complexes of β-cyclodextrin-NH_2_ with adamantane.

***Synthesis of Supramolecular polysaccharide nanotheranostics (SPN):***The supramolecular polysaccharide nanotheranostics were synthesized by facilitating the electrostatic interactions between positively charged β-cyclodextrin-NH_2_-complexes and the negatively charged sodium alginate backbone. These interactions along with the aromatic interactions between kinase inhibitor molecules which are in close proximity, facilitates the formation of supramolecular nanoassembly. The ratio of the PI103- β-cyclodextrin complex and polymer construct (activatable imaging probe modified alginate polymer) in the nanoassembly was carefully optimized to be 1:1 such that it forms stable nanostructures. Typically, 2 mg of polymer construct and 2 mg of PI103- β-cyclodextrin complex results in the formation of supramolecular polysaccharide nanotheranostics (SPN). The amount of kinase inhibitor encapsulated in the SPNs was calculated to be 90 ug/mg of kinase inhibitor with respect to polymer as measured by absorbance spectroscopy. Similarly, a control nanotheranostics were synthesized using β-cyclodextrin-NH_2_ (without adamantane kinase inhibitor conjugate) and polymer construct and referred as “SPNC” (supramolecular polysaccharide nanotheranostics control). Also, fluorescent control polysaccharide nanoassemblies were synthesized where the imaging probe was designed not to have a quencher molecule and thus can be used as fluorescent supramolecular polysaccharide nanotheranostics (“always on” system) and these are referred as “SPNF”.

**Figure 3:**
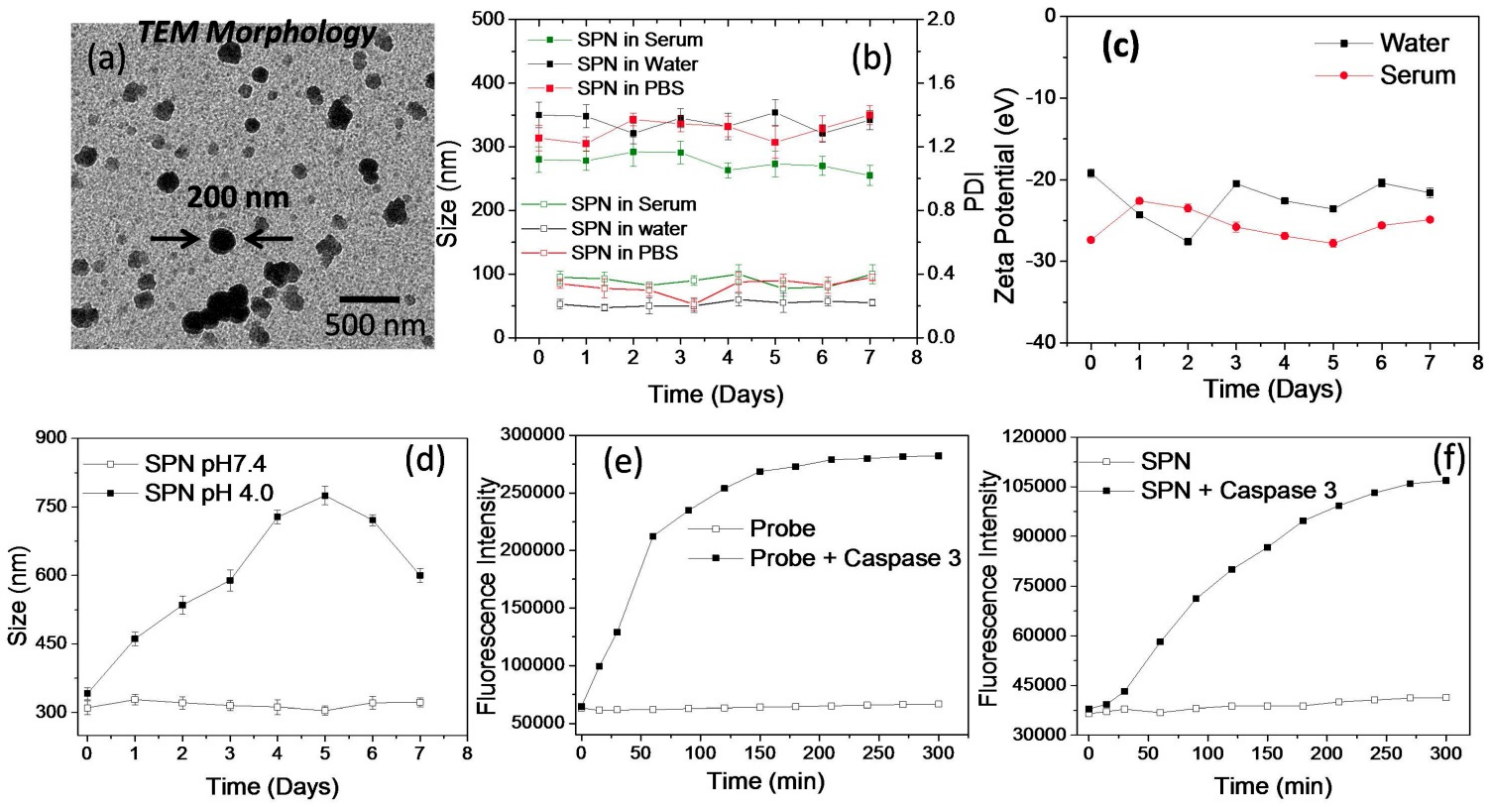
** Physiochemical characterization and enzyme susceptibility of SPN:** High resolution transmission microscopic image of the nanoparticles (a), plot showing DLS size and PDI of the SPN in human serum, water and phosphate buffer saline at pH 7.4 (b), plot showing the zeta potential of the SPN in water and human serum (c), plot showing pH responsive disassembly of the SPN's based on the size measurement using DLS technique (d), plots showing the fluorescence recovery from the activatable probe in the presence and absence of caspase 3 enzyme (e) and plots showing the fluorescence recovery from the SPN's in the presence and absence of caspase 3 enzyme (f).

***In vitro characterization and enzyme susceptibility studies of SPN's:*** The SPNs are aimed to deliver PI3K inhibitor to the cancer tissues and hence the formation of appropriate size of self assembled structures is necessary to facilitate its accumulation in the tumor tissues. These SPNs were also designed to deliver kinase inhibitor function responsive activatable imaging probe. The co-delivery of kinase inhibitor (PI103) and the activatable imaging probe from the same nanoassemblies could ensure enhanced sensitivity and selectivity in imaging inhibitor response. The aqueous self-assembly of these polysaccharide derivatives resulted in the formation of SPN's with hydrodynamic diameter of around 250 ± 50 nm as determined by DLS technique. The morphology of the SPN's was analyzed by transmission electron microscopy (TEM) (**Figure 3a**). Size and morphology obtained from the TEM analysis was in accordance with result obtained from the DLS techniques and thus validates the formation of SPNs of uniform size. Next, the stability of the polysaccharide derivatives was studied in aqueous medium using dynamic light scattering (DLS) technique (**Figure 3b**). The stability of these SPNs was evaluated in the physiologically relevant conditions such as phosphate buffer saline (pH 7.4) and human serum. The hydrodynamic diameter and polydispersity index of these SPNs were monitored for over 7 days. As shown in **Figure 3b**, the SPNs maintained the structural stability in these mediums confirms that these formulations are very stable in all the three mediums. The zeta potential of these SPN's was studied using DLS and the plot is shown in **Figure 3c**. The zeta potential value was found to be -22 ± 5 eV which facilitate the easy redispersion of SPN's and accounts for their high colloidal stability. To study the effect of the acidic pH (mimicking tumor conditions) on the stability and disintegration of the SPN's, an *in vitro* experiment was designed where SPN's were incubated in pH 4.0 buffer and the stability was studied by measuring hydrodynamic diameter of the SPN's. As shown in **Figure 3d**, the hydrodynamic diameter of the SPN changes over a period of time and in 7 days they were found to be destabilized in the acidic condition. On the other hand, the SPN's at pH 7.4 in water showed a superior stability. The susceptibility of activatable imaging probe for the enzymatic cleavage is essential to better monitor the kinase inhibitor efficiency and hence an *in vitro* experiment was performed to examine the activation of the imaging probe when incubated with activated enzyme. The GK-DEVD-APC activatable imaging probe with fluorophore and quencher molecule was incubated with recombinant caspase-3 enzyme and enzymatic activity was recorded as function of fluorescence intensity over time. In the absence of the enzyme, the activatable imaging probe did not show any emission signal as the fluorescence of the chromophore was quenched by quencher molecule. The enzymatic activity cleaves off the peptide sequence to activate the fluorophore signal. The plot of activatable probe- enzyme activity shows the increase in the fluorescence intensity with increase in time whereas the probe alone (without caspase 3 enzyme) did not show any enhancement in the fluorescence signal (**Figure 3e**). These results demonstrate the susceptibility of the imaging probe to cleave under enzymatic conditions. To study the similar effect of enzyme activity on SPN's, they were incubated with recombinant caspase 3 enzyme and the increase in fluorescence signal was monitored over time. As shown in **Figure 3f**, the consistent increase in the fluorescence intensity clearly reveals the ability of the enzyme to penetrate SPN's to cleave off the peptide sequence. Under similar condition, SPN's without caspase-3 enzyme did not show any fluorescence enhancement and thus validating the necessity of the enzymatic activity to better monitor the imaging signal. These results show that release of caspase-3 enzyme is necessary to monitor the imaging signal and this system can be used to monitor the kinase inhibitor mediated caspase 3 enzyme activity n tumor cells in real time.

**Figure 4:**
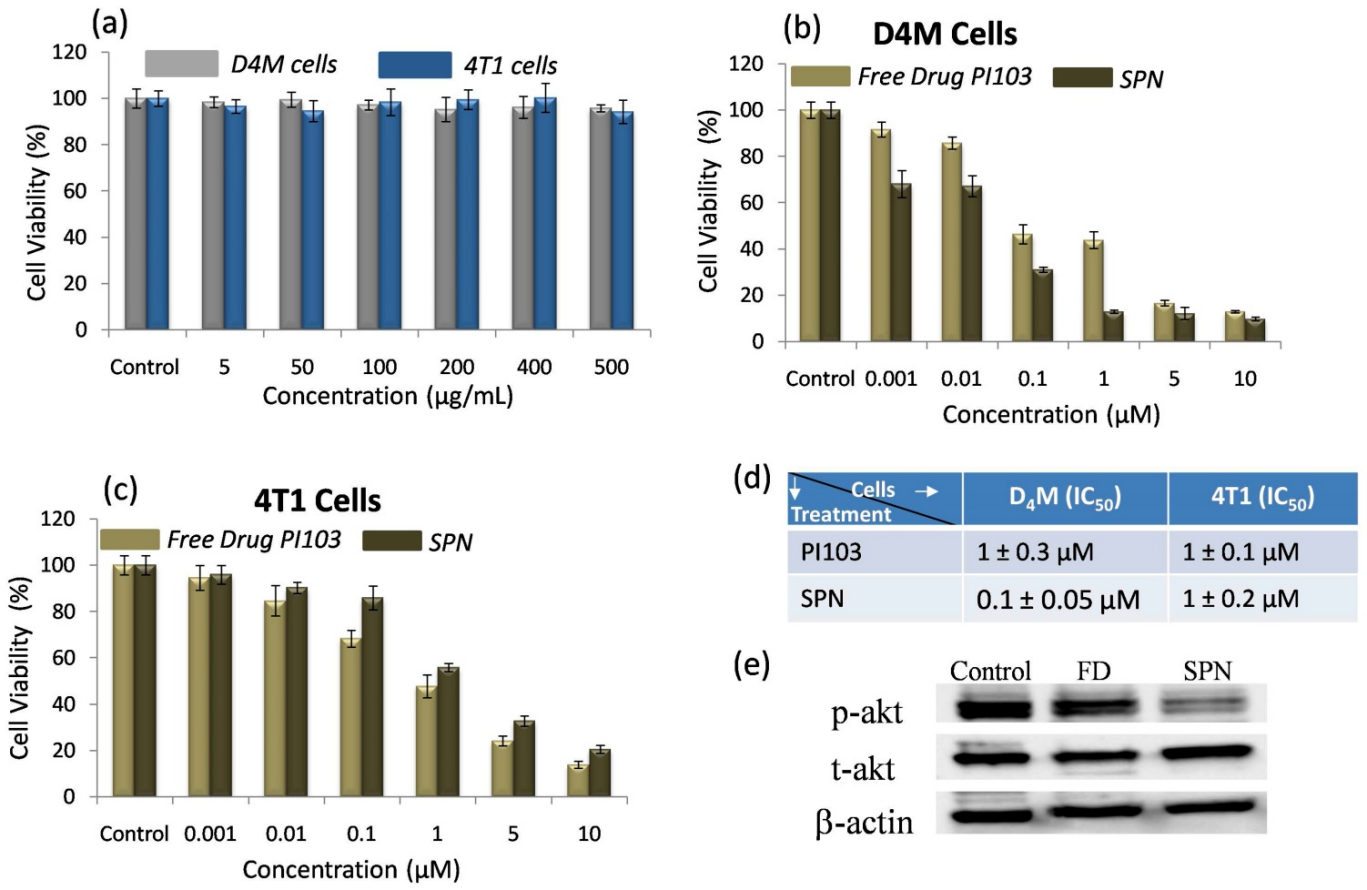
** In vitro cytotoxicity and western blot analysis of SPN:** Graph showing the effect of free SPN concentration on the cell viability of D4M and 4T1 cells (a), graph showing the cytotoxic effect of free PI103 and SPN on D4M cells (b), graph showing the cytotoxic effect of free PI103 and SPN on 4T1 cells (c), table depicting IC_50_ values for free kinase inhibitor and SPN (d) and representative western blot shows the expression of Phospho Akt and Total Akt in D4M cells after 12 hours of treatment with free PI103 and SPN (e). For cell viability studies, the SPN's were incubated with cells for 48 hours.

***In vitro cytotoxic efficacy of SPN's:***Despite PI3K signaling pathways being hyperactive in many cancers, PI3K inhibitors have shown limited efficacy in terms of long-term tumor growth inhibition. To enhance therapeutic efficacy of PI3K inhibitor, SPNs were designed which can deliver kinase inhibitor to the target site to efficiently inhibit the pathways in cancer cells and activate the apoptotic pathways. First, *in vitro* cytotoxic effects of the SPN's were studied in BRafV^600E^ melanoma (D_4_M) and triple-negative breast cancer (4T1) cells with activated PI3K signaling. The cells were incubated with blank SPN's (without PI3K inhibitor) and their cytotoxic effects were studied using MTT assay. As shown in **Figure 4a**, blank SPNs alone did not induce any cytotoxic effects in both the cell types (D_4_M and 4T1) demonstrating that this vehicle system can be used to monitor PI3K inhibitor activity. To assess the cytotoxic effects of free kinase inhibitor (PI103) and kinase inhibitor loaded SPN's in D_4_M and 4T1 cells, these cells were incubated with either PI103 or SPN's for 48 hours and the cell viability was analyzed by MTT assay (**Figure 4b and c**). From the data in **Figure 4b**, it was confirmed that in case of D_4_M cells, kinase inhibitor encapsulated SPN's showed a better cell killing than free kinase inhibitor whereas in case of 4T1 cells, SPN's showed a similar performance as that of free kinase inhibitor. The IC_50_ values demonstrates the efficiency of these SPN's in both D_4_M and 4T1 cells (**Figure 4d**). To further confirm the inhibition of phosphorylation of PI3K and downstream Akt signaling in cancer cells by SPN, western blot analysis was carried out where D_4_M cells were treated with 5 μM concentration of free PI103 or SPN. From the western blot analysis (**Figure 4e**), it was confirmed that the SPN induced significant inhibition of phosphorylation of Akt as compared to free PI103. This lower efficacy could be due to rebound increase in phosphorylation of Akt in case of free kinase inhibitor PI103, which was shown in recent studies^51^. To study the effect of free PI103 inhibitor, adamantane-PI103 conjugate and SPN on phosphorylation at earlier time points, a time dependent treatment with D4M cells was carried out and the obtained western blot data is given in **Supplementary Figure 11**. This data proved the fact that, at earlier time point (4 hours), free PI103 inhibitor, adamantane-PI103 conjugate and SPN could inhibit the phosphorylation but as time increases (8 and 12 hours) only SPN showed a better inhibition of phosphorylation as compared to free PI103 and adamantane-PI13 conjugate. This proves that rebound increase in phosphorylation of Akt was observed for free PI103 and adamantane-PI103 conjugate and hence it is indeed necessary to deliver the PI103 using a nanocarrier approach. Also, the adamantane-PI103 conjugate did not showed any other off target effects such as increased or decreased target protein binding or steric hindrance for binding the target protein. Thus, the use of SPN's in the present approach facilitates the delivery of the kinase inhibitor in cancer cells to accomplish better therapeutic efficacy.

**Figure 5:**
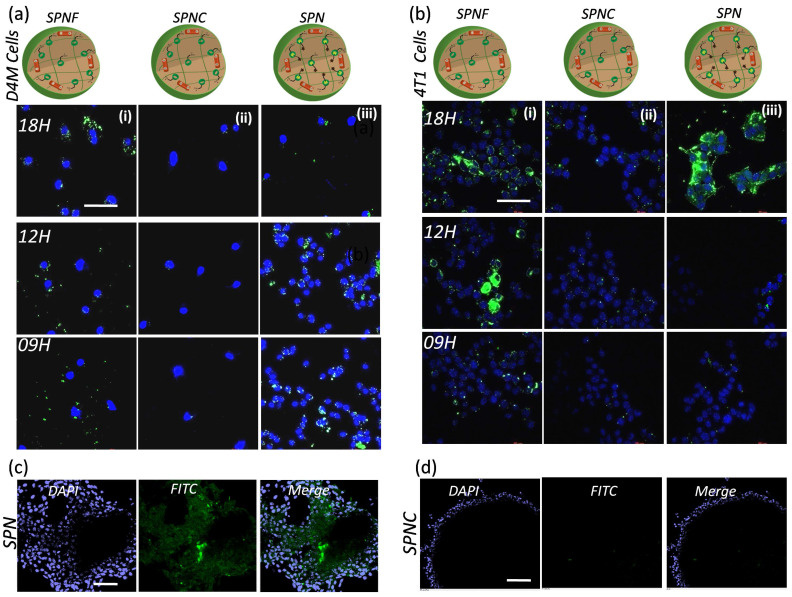
** Efficiency studies of SPN in cancer cells and tumor spheroids:** Time dependent confocal microscopic images showing the intracellular kinase inhibitor action mediated fluorescence turn on from the SPN in D4M cells (a), and in 4T1 cells (b). The first, second and third column in the picture (a) represents the cellular uptake of SPNF, SPNC and SPN. The same trend follows in the picture (b) as well. The nuclei are stained with DAPI. The concentration of the kinase inhibitor used is 5uM and the incubation time is as mentioned in the images. Scale bar in the images is 20 μm, confocal microscopic images of nanoparticles accumulation in the 4T1 tumor spheroids for SPN (c) and for SPNC (d). The SPN's were incubated for 12 hours with tumor spheroids followed by fixing and imaging. The nuclei are stained with DAPI. The concentration of the kinase inhibitor used is 5uM. The scale for the images c and d is 100μm.

***In vitro efficacy of SPN's to monitor the kinase inhibitor response:***The nanotheranostics system that can deliver the kinase inhibitors to the tumor site while simultaneously detecting the inhibitor response in action can have a major impact on targeted therapies. Here, we have developed a SPN system to achieve tumor specific pH responsive kinase inhibitor release that induces cancer cell death and apoptotic enzyme sensitive activatable probe that enables the real time monitoring of the kinase inhibitor response. To examine the efficacy of the SPN's in imaging the response, D_4_M and 4T1 cancer cells were incubated with activatable probe and control nanotheranostics (**Figure 5**). First, D_4_M cells were incubated with SPNF, SPNC and SPN's and the activation of the fluorescence signal from 5FAM dye was imaged at different time points to study the effect of the kinase inhibitor response. As shown in **Figure 5a (i)**, the cells treated with SPNF showed appearance of the green emission corresponding to 5FAM dye which confirmed the increase in cellular internalization of the nanotheranostics at various time points. The appearance of the green emission from the SPNF was expected as these were designed as “always on” nanotheranostics. Thus, fluorescent intensity from SPNF's accounts for the maximum signal that could be achieved using SPN system and used as positive control to monitor the activation of fluorescent signal from SPN. When similar treatment was carried out for SPNC, the cells did not show fluorescent signal at any time point which confirmed that the 5FAM dye remained in the quenched state due to the presence of the dye in the proximity of the quencher molecule. Since the SPNC were designed without the kinase inhibitors, the absence of the emission signal was anticipated and validates that there was no non-specific activation of the imaging probe in the cancer cells (**Figure 5a (ii)**). The representative confocal microscopic images from **Figure 5a (iii)**, show the time-dependent increase in the fluorescent signal from SPN in the cancer cells, which accounts for the activation of the 5FAM dye due to caspase-3 mediated peptide cleavage. This proves that the SPNs can activate the apoptosis machinery and caspase 3 enzyme necessary for the peptide cleavage to activate the probe. Similar results were observed in aggressive breast cancer cells (4T1) and the representative confocal microscopy images are shown in **Figure 5b (i), (ii), and (iii).** These studies confirmed that the SPN system can deliver kinase inhibitor inside the cancer cells and facilitates activation of the apoptosis machinery that can be monitored via imaging. To further prove the fact that SPN releases the PI103 inhibitor inside the cells to activate the apoptotic machinery, the PI103 concentration in SPN treated cells (D4M) at different time-points (4,8 and 12 hours) were calculated by lysing the cells and measuring the absorbance of the PI103 and data is showed in **Supplementary Figure 12.** This experiment proved that with increasing the time, SPN could increase the concentration of the PI103 inside the cells which facilitates the apoptosis process. Moreover, the feasibility of the SPN's to work with multiple cancer cell lines also makes it more desirable to be used as theranostics for multiple cancer types where kinase signaling is overactivated.

***Monitoring the efficacy of SPN's in 3D in vitro models:*** We next examined the sensitivity of the SPN's in 3D tumor models, as 3D tumor spheroids better mimic the *in vivo* conditions of the tumor microenvironment. The 4T1 tumor spheroids were grown and incubated with either the SPN's or SPNC treatment. The tumor spheroids were imaged 12h after the treatments. As shown in **Figure 5c**, the appearance of the green fluorescent signal in the intracellular environment in the spheroids confirms the activation of apoptotic machinery which releases caspase 3 enzymes and activates the fluorescent dye. Interestingly, when tumor spheroids were treated with SPNC (without kinase inhibitors), there was no activation of the fluorescent signal even inside the necrotic core of the tumor spheroids (**Figure 5d**). These results demonstrate the ability of the SPN's to penetrate into the tumor spheroids and the response can even be monitored at depth of 20μm. Thus, the current approach establishes a platform for targeted therapies to arrest the tumor growth in 3D tumor models and to monitor real time kinase inhibitor response.

**Figure 6:**
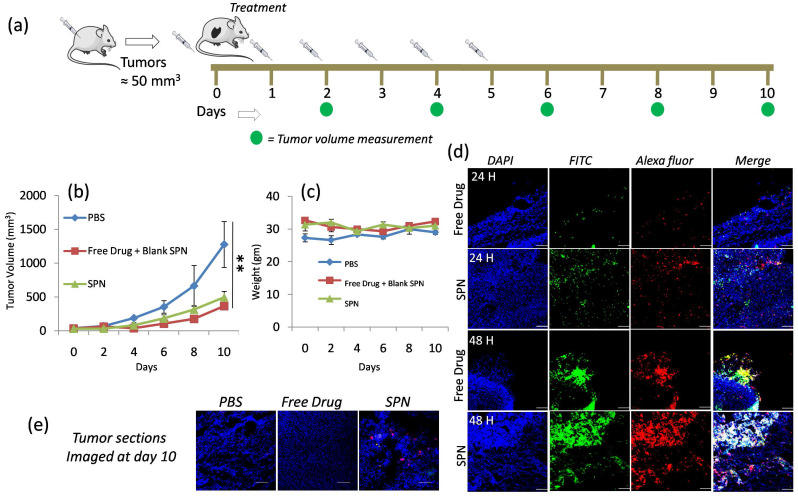
** In vivo efficacy of SPN in D4M model:** Schematics showing the course of injection of SPN given to D4M tumor bearing mice (a), graph shows effect of different group treatments on tumor growth inhibition (b), graph shows the weight of the mice during SPN treatment (c), representative confocal microscopic images of the tumor sections of the excised tumor from the mice at 24h and 48 h time point (d) and confocal microscopic images of tumor section of the excised tumor after 10 days of treatment (e). To locate the cleaved caspase-3, the tumor sections were stained with primary rabbit anti-mouse cleaved caspase-3 antibody followed by Alexa Fluor 594 goat anti rabbit IgG secondary antibody. The nuclei were stained with DAPI. The scale bar in images is 100μm.

***In vivo* efficacy studies in D_4_M Melanoma Model:** To study the *in vivo* efficacy of SPNs in D_4_M melanoma model, D_4_M cells were injected subcutaneously into the right flank of the C57B/L6 mice and tumor were allowed to grow. Once the tumor reached the desired size (50mm^3^), mice were differentiated into three groups and treatment was given as (I) phosphate buffer saline, (II) free kinase inhibitor + blank SPN (fluorescently labeled and without kinase inhibitor) and (III) SPN with kinase inhibitor (PI103) as shown in schematics in **Figure 6a**. The tumor growth inhibition was monitored by measuring the tumor volumes and the data shown in **Figure 6b**. This data clearly reveals the inhibition of tumor growth in the group treated with SPN compared to the control group (PBS injection) and comparable to free PI103 administration. The body weights of the mice were observed throughout the treatment course and the plot in **Figure 6c** reflects the consistency in the body weight of the mice. This suggests that the treatments did not induce any toxicity to the mice and results obtained were purely based upon the therapeutic action of the kinase inhibitor delivered at the target site. To understand the efficacy of the SPN in inducing the apoptosis through activation of caspase-3 enzyme, an *ex vivo* imaging experiment was designed where mice were given the SPN treatment and the tumors were isolated from the mice at 24- and 48-hour time points. These tumors were sectioned and stained with primary rabbit anti mouse cleaved caspase-3 antibody followed by Alexa Fluor 594 goat anti rabbit IgG secondary antibody. The confocal images for the tumor sections were shown in **Figure 6d**, the nuclei in the tissues were stained with DAPI and the activation of the cleaved caspase-3 can be monitored by red color from the dye-labeled antibody. As shown in **Figure 6d**, 48-hour time point showed significant fluorescence activation as compared to 24 hours. The increase in the green and red fluorescence in the Figure 6d reveals the activation of caspase-3 followed by the cleavage of peptide sequence to turn on the green signal. This experiment proves that effectiveness of the current system to monitor the kinase inhibitor action through the imaging. The close observation of the 48-hour time point images revealed that the activation of fluorescence signal was better in case of SPN treated groups where the kinase inhibitor and imaging probes were delivered using the same nanoparticles system. In case of free kinase inhibitor + blank SPN (has imaging probe but no Pi103 inhibitor) group, the blank SPN and free kinase inhibitor were given separately and hence showed poor signal activation. This observation proves the importance of synchronized delivery of the activatable probe and kinase inhibitor to better monitor the activation signal. The yellow color (overlap of green and red) in the merged image of SPN at 48-hour time point clearly reveals the better activation of the fluorescence signal which is significantly higher as compared to the free kinase inhibitor. Further to confirm the efficacy of the SPN in activating the apoptosis, tumors from each group at the end of day 10 were harvested, sectioned and stained with antibody to study the caspase-3 activation (**Figure 6e**). The presence of red and yellow color in the merge image of SPN confirmed the activation of the caspase even after day 10. This proves the ability of the SPN to release the kinase inhibitor in a sustained manner for longer time point to accomplish better therapeutic efficacy. This fluorescence activation was not seen in case of free kinase inhibitor and PBS group. This kinase inhibitor action responsive monitoring of the fluorescence signal in the tumor sections also proved the fact that these SPN could home the tumor tissues using the EPR effect to release the loaded cargoes. To study the cytotoxic effects of these SPN on different organs in mice after the administration of different treatments groups, we have examined the tissue sections with TUNEL assay, and the data is provided in **Supplementary Figure 13**. To carry out this experiment, the liver and kidney sections were stained with TUENL (red) stain and the nuclei were stained with DAPI. The representative confocal images showed in Supplementary Figure 13 confirmed that these SPN and free PI103 did not induce any toxic effects to liver whereas minimal toxicity was observed in kidney. Although some toxicity was observed in the kidney from SPN and free PI103, the results obtained in the present study confirmed that these SPN's induces better cytotoxic effects in the tumors (see figure 6d) and also enables the monitoring of apoptosis through imaging. Overall, these observations demonstrate that the SPN system can potentially be used for targeted therapies as a nanotheranostics system in biomedical field.

## Conclusion

In conclusion, we have developed a two-stage supramolecular polysaccharide nanotheranostics (SPN) system that induces significant kinase inhibition and facilitates caspase-mediated apoptosis of the cancer cells. SPNs also enable fluorescence tracking of the kinase inhibitor action and allow real-time monitoring of the therapeutic efficacy. The naturally occurring polysaccharide sodium alginate was modified with activatable imaging probe to synthesize the polymer construct. The β-cyclodextrin and adamantane-kinase inhibitor conjugate inclusion complex was synthesized and incorporated into newly designed polymer construct using supramolecular chemistry approach. The SPNs were found to be very stable at physiologically relevant conditions and have been successfully employed to deliver the kinase inhibitor to the tumor. The integration of the activatable probe along with the kinase inhibitor in the present approach allowed us to monitor the kinase inhibitor action using fluorescence imaging. This was also validated in the aggressive breast 3D tumor spheroids where SPNs enabled the delivery of the kinase inhibitor molecules at core of the spheroids. Further, the efficacies of these SPN were examined *in vivo* in D_4_M melanoma model and showed significant tumor growth inhibition as compared to control groups. The fluorescence activation of these SPN was studied by *ex vivo* imaging of the tumor sections. The *ex vivo* imaging confirmed the homing of these SPN in tumor microenvironment and enhanced fluorescence activation was observed as a result of the kinase inhibitor action. Overall, the present design has a potential to deliver the small molecule inhibitors to tumor and monitor its action through imaging. This modular system can be adapted to different molecular targeted therapies and various cancer types as well as can be used to monitor the action of combination therapies such as combination of targeted therapies with chemotherapy or immunotherapy.

## Supplementary Material

Supplementary figures.
