# Supplementary material for: Supramolecular Polysaccharide Nanotheranostics that Inhibit Cancer Cells Growth and Monitor Targeted Therapy Response
Source: Nanotheranostics. 2020 May 18;4(3):156–72. doi: 10.7150/ntno.44703 (PMC7256014; doi:10.7150/ntno.44703)
Supplement: Supplementary file 1 — Supplementary figures. [file ntnov04p0156s1.pdf]

Supplementary Material

Supramolecular Polysaccharide Nanotheranostics that Inhibit Cancer Cells Growth and Monitor Targeted Therapy Response

Nilesh Deshpande[1], Anujan Ramesh[2], Dipika Nandi[3], Anh Nguyen[1], Anthony Brouillard[1] and Ashish Kulkarni[1,2,3,4,*]

[1]Department of Chemical Engineering, University of Massachusetts, Amherst, MA, USA;
[2]Depatment of Biomedical Engineering, University of Massachusetts, Amherst, MA, USA;
[3]Department of Veterinary and Animal Science, University of Massachusetts, Amherst, MA, USA;
[4]Center for Bioactive Delivery, Institute for Applied Life Sciences, University of Massachusetts, Amherst, MA, USA;

*Corresponding Author: akulkarni@engin.umass.edu

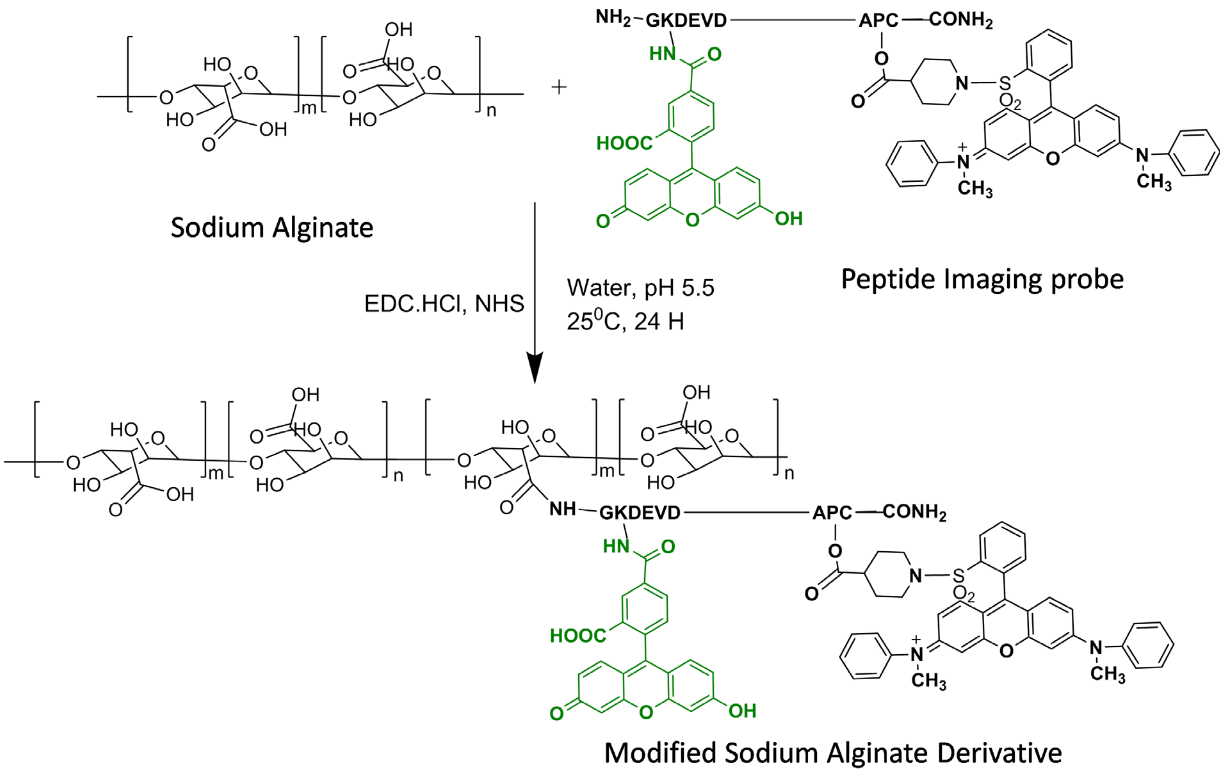

Supplementary Figure 1: Synthetic scheme for sodium alginate modification with peptide imaging probe.

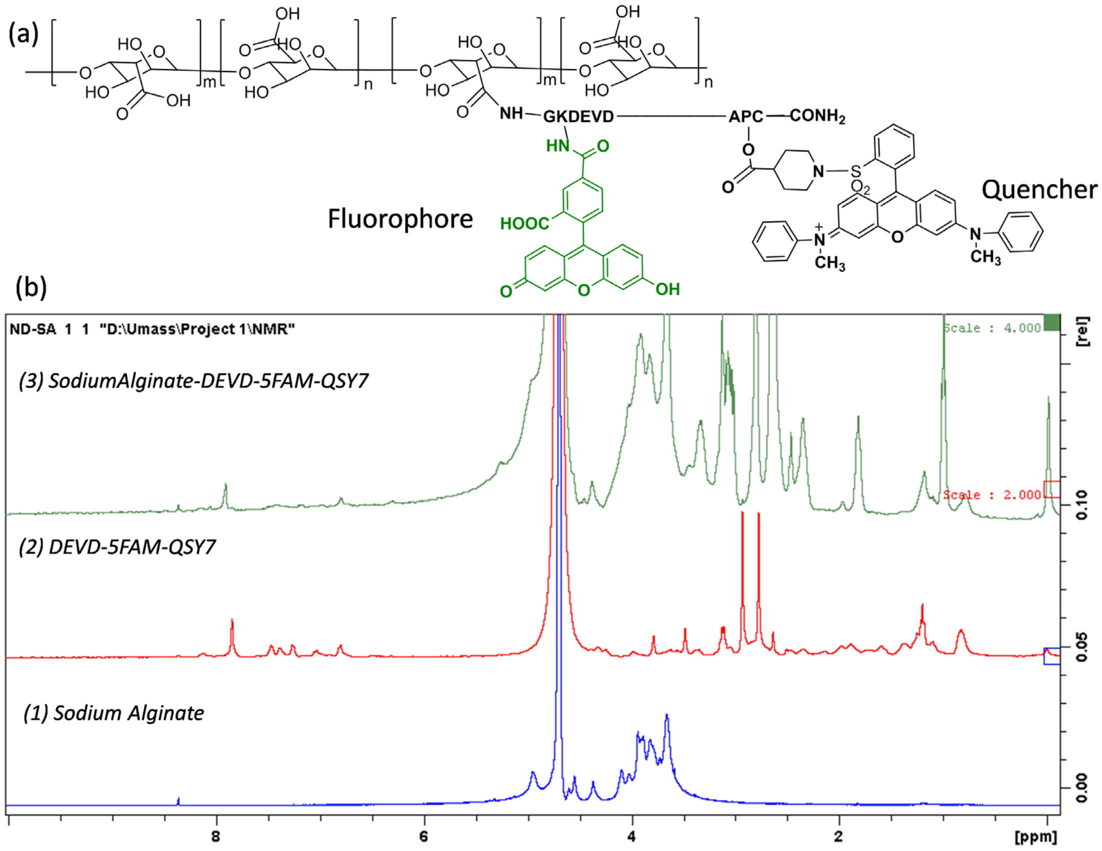

Supplementary Figure 2: Chemical structure of sodium alginate modified with DEVD peptide sequence (a), ^{1}H NMR of sodium alginate (1), peptide sequence (2) and modified sodium alginate (3) (b).The peptide sequence is equipped with a 5FAM fluorophore and a quencher molecule (QSY7) which induces fluorescence quenching when present in the close proximity. The ^{1}H NMR is recorded in deuterated water as a solvent.

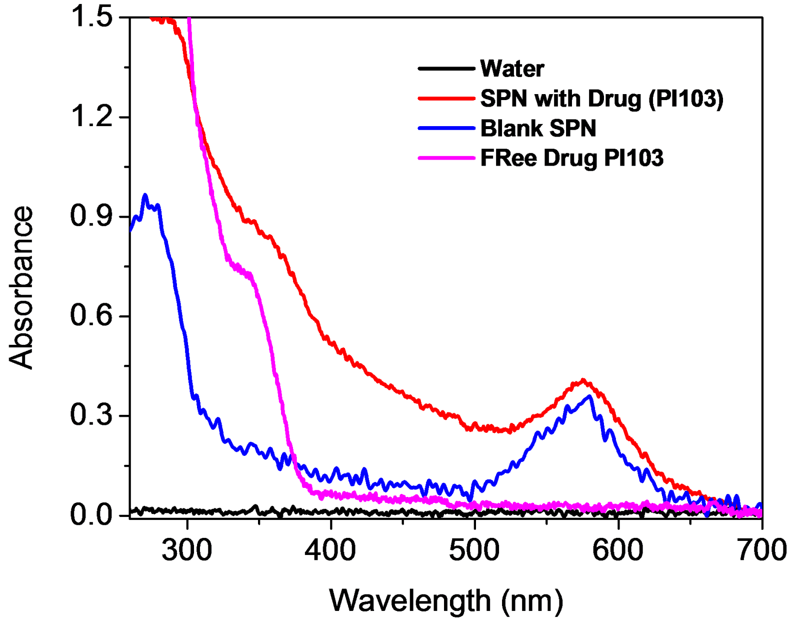

Supplementary Figure 3: Absorbance plot for SPN with drug, blank SPN and free drug PI103. The equation used for calculation of amount of drug/ dye are as follows,

1. Dye Y = 0.101 X + 0.067   2. For Drug Y = 0.089 X + 0.041

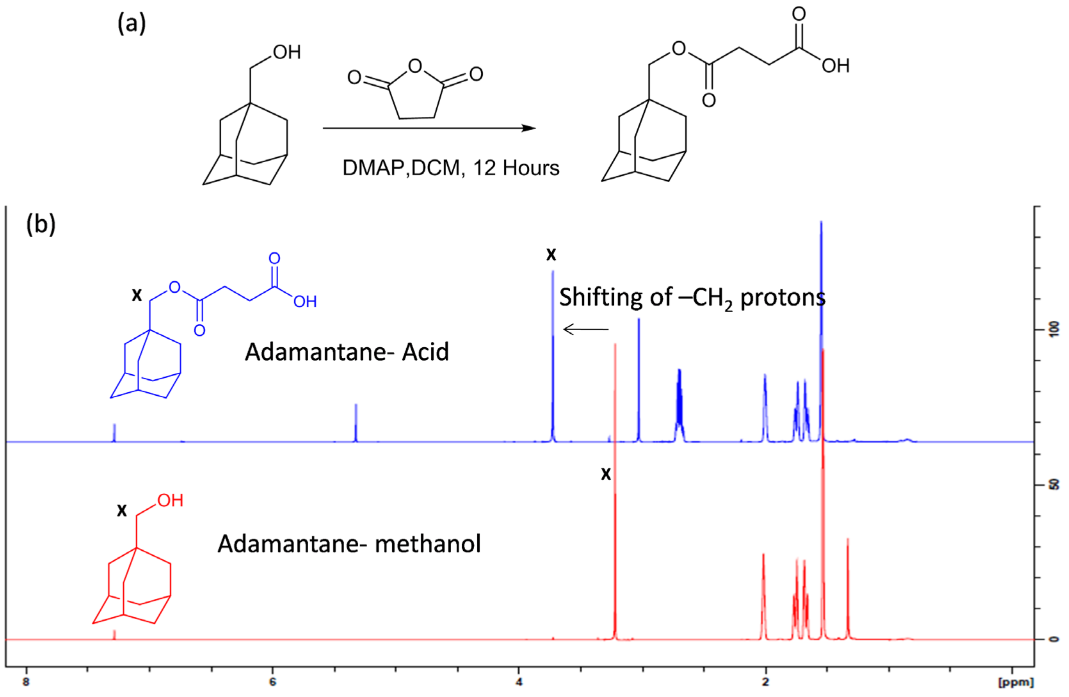

Supplementary Figure 4: Synthetic scheme for adamantane-acid synthesis (a) and ^{1}H NMR for adamantane methanol and adamantane-acid (b).

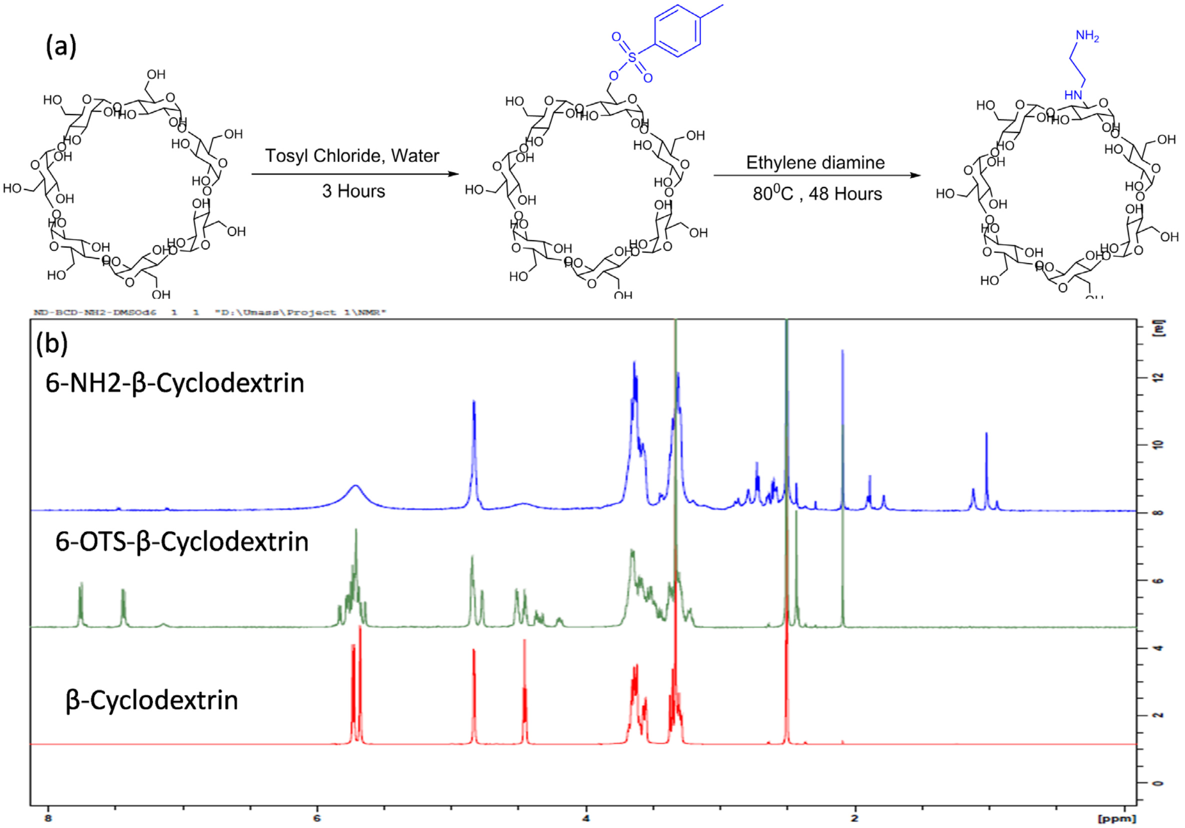

Supplementary Figure 5: Synthetic scheme for β–CD-NH_2 Synthesis(a) and ^{1}H NMR for β-Cyclodextrin, 6-OTS-β-Cyclodextrin and 6-NH2-β-Cyclodextrin (b).NMR were recorded in dmso-d6.

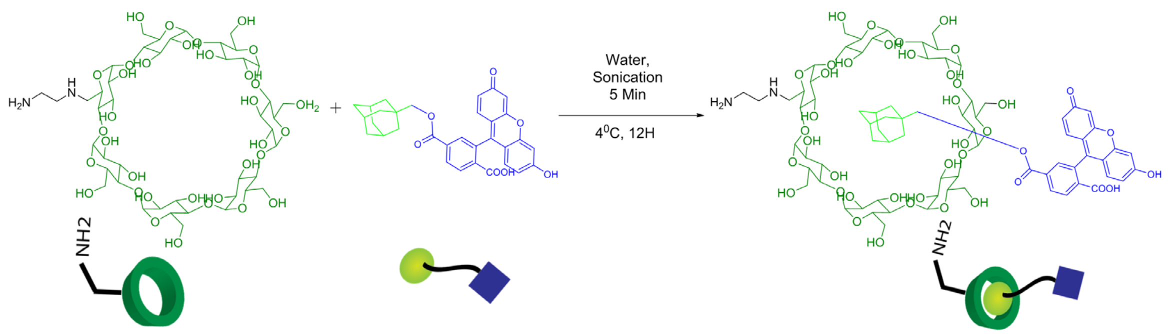

Supplementary Figure 6: Chemical reaction showing inclusion complexation between β-CD-NH_2 and adamantane-FITC conjugate.

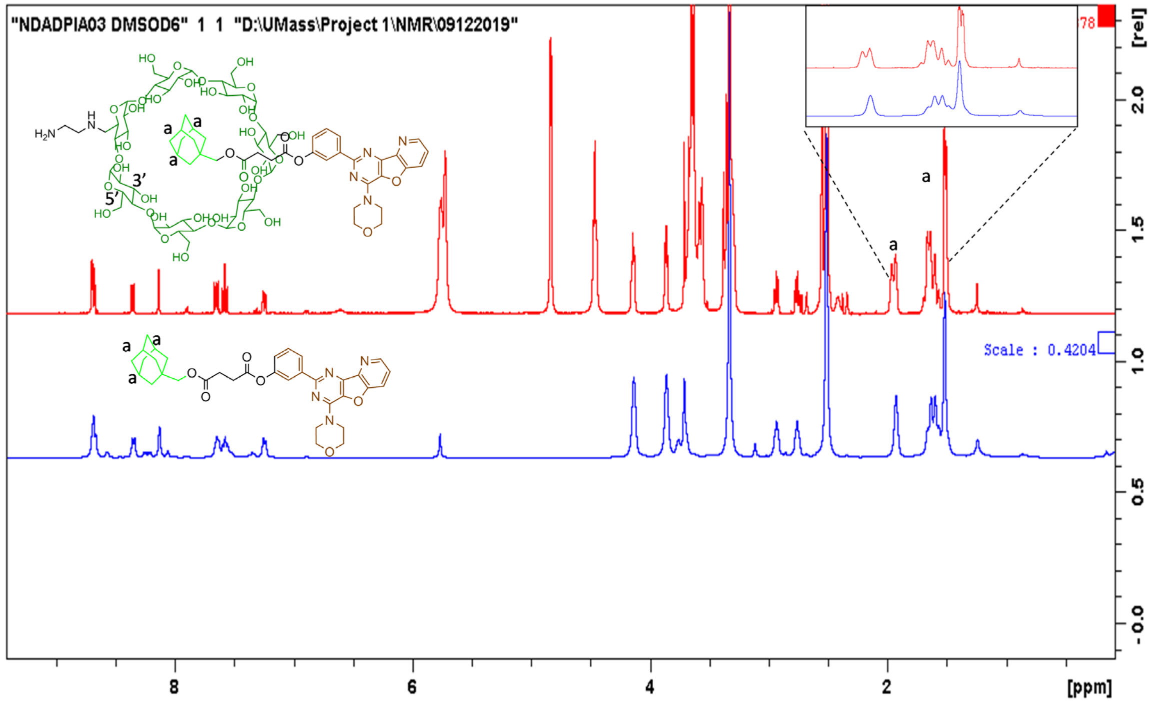

Supplementary Figure 7: ^{1}H NMR Spectrum of adamaantane-PI103 conjugate (a) and β- CD-NH2-AD-PI103 inclusion complex (b).

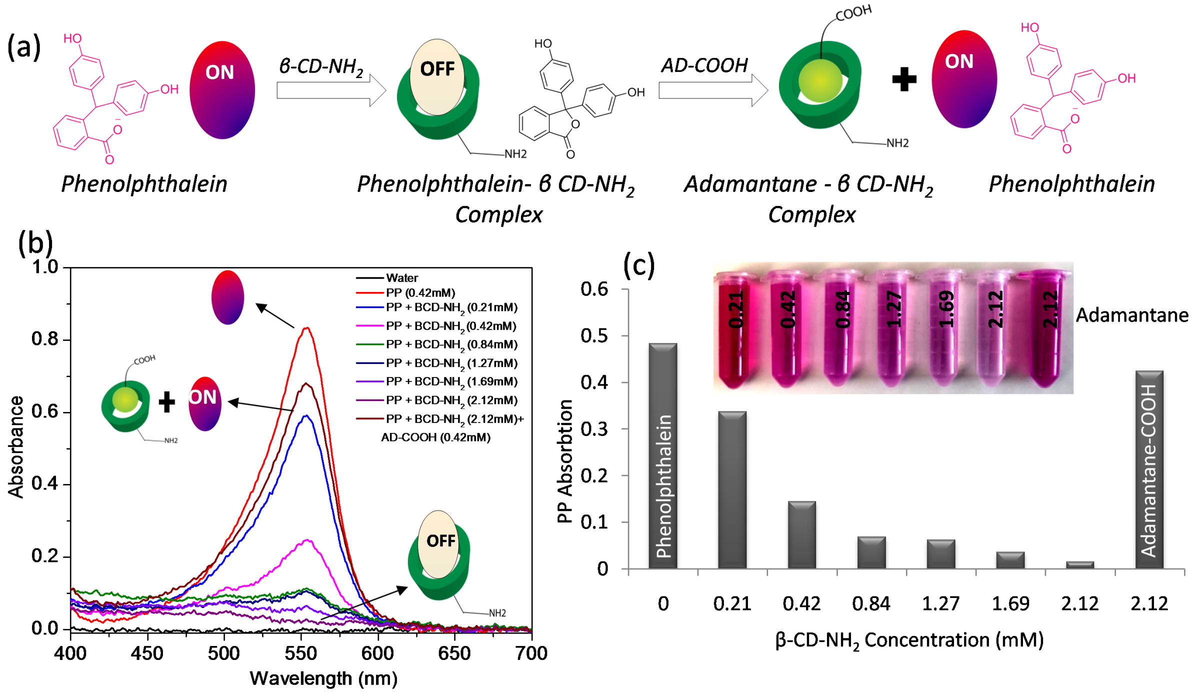

Supplementary Figure 8: Schematics illustrating the phenolphthalein complexation and decomplexation phenomenon with β-CD-NH_2 (a), absorbance spectrum of phenolphthalein in the presence of different concentration of β CD-NH_2 (b) and the plot of absorbance intensity versus concentration of β CD-NH_2 (c). Phenolphthalein occupies β CD-NH_2 core and converts to its colorless lactone form and upon replacement by adamantane, regains its pink color. The experiment is carried out in aqueous solution having pH 10.5.

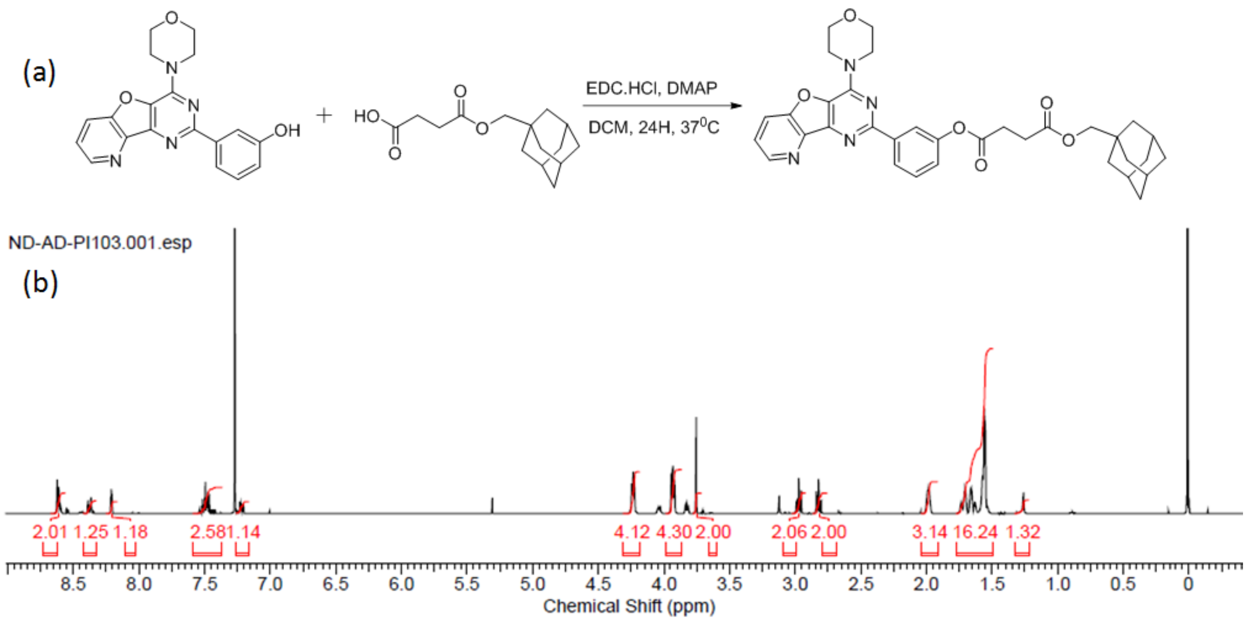

Supplementary Figure 9: Synthetic scheme for AD-SA-PI103 (a) and ^{1}H NMR for AD-SA-PI103 (b).

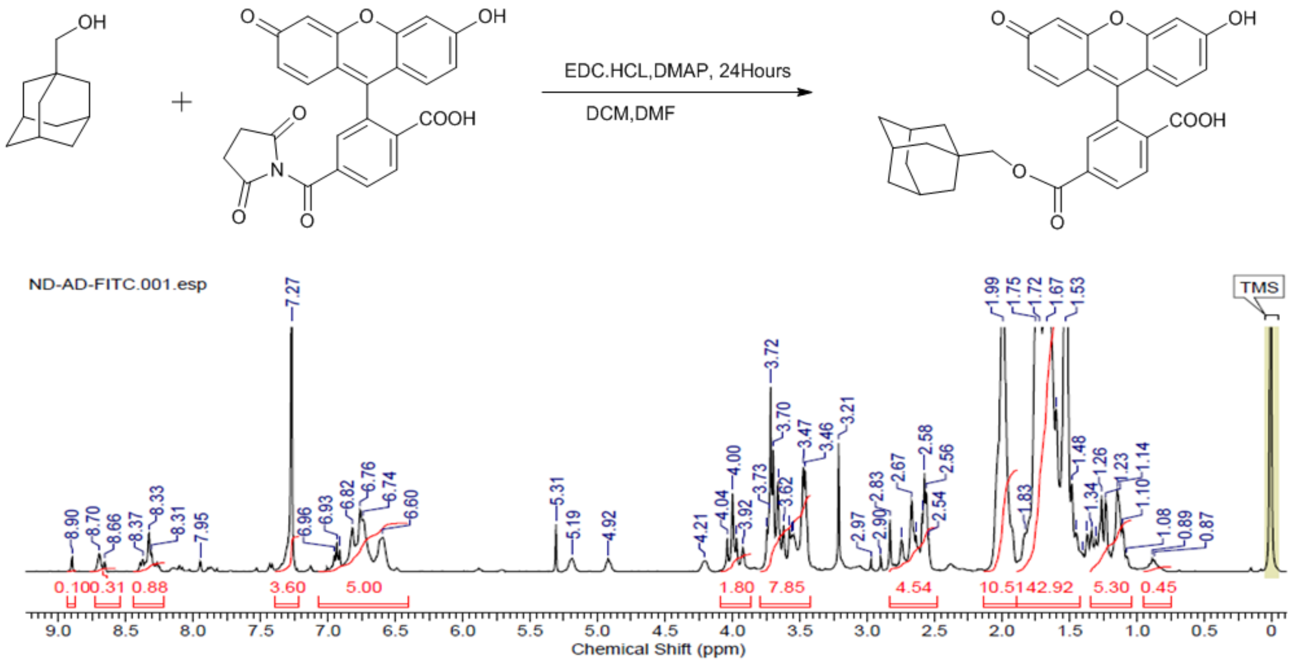

Supplementary Figure 10: Synthetic scheme for AD-FITC (a) and ^{1}H NMR for AD-FITC (b).

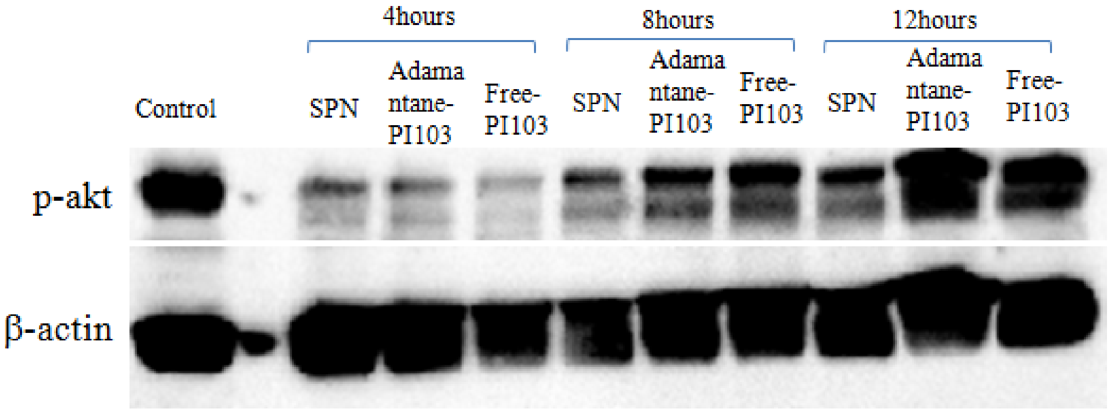

Supplementary Figure 11: Representative western blot shows the expression of Phospho Akt in D4M cells after 4, 8 and 12 hours of treatment with free PI103, adamantane-PI103 conjugate and SPN.

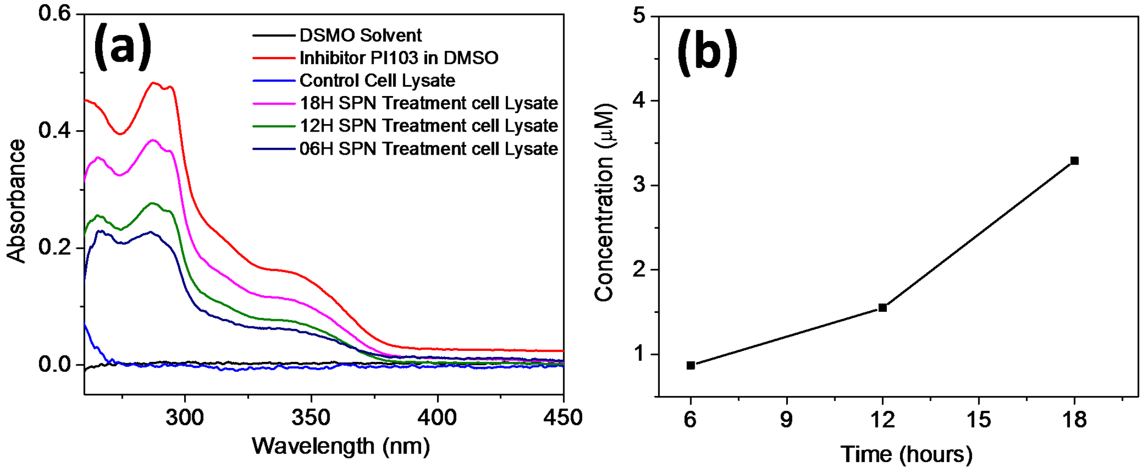

Supplementary Figure 12: Absorbance plot for DMSO solvent, PI103 inhibitor in DMSO, control cell lysate and SPN treated cell lysates at different time points (a), a plot showing increase in the concentration of PI103 in D4M cells with respect to time (b). The concentration is calculated by measuring the absorbance of the cell lysate.

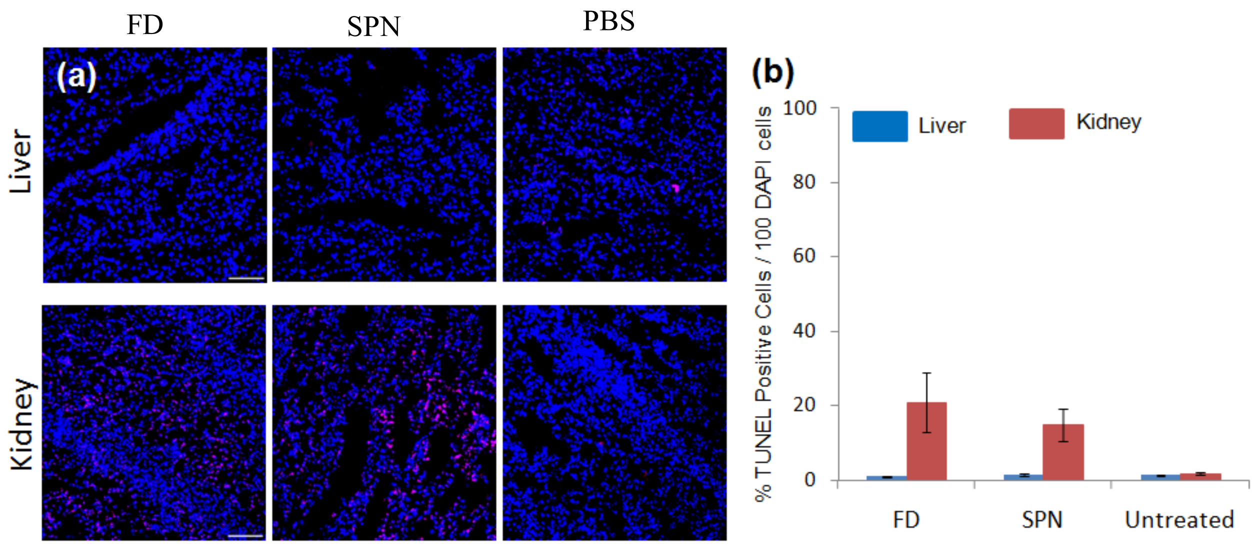

Supplementary Figure 13: Representative confocal images of tissue sections from liver and kidney of the animals treated with different treatment groups (a). The sections were labeled for apoptosis using TUNEL (red) stain and counterstained with DAPI (Blue), graph shows the quantification of apoptosis from tissue sections as a percentage of TUNEL + Cells per 100 DAP cells (b). Scale bar : 100 µm.
